# Population genomics of wild and laboratory zebrafish (*Danio rerio*)

**DOI:** 10.1111/j.1365-294X.2011.05272.x

**Published:** 2011-10

**Authors:** Andrew R Whiteley, Anuradha Bhat, Emilia P Martins, Richard L Mayden, M Arunachalam, Silva Uusi-Heikkilä, A T A Ahmed, Jiwan Shrestha, Matthew Clark, Derek Stemple, Louis Bernatchez

**Affiliations:** * Department of Environmental Conservation, University of MassachusettsAmherst, MA 01003, USA; † Institut de Biologie Intégrative et des Systèmes (IBIS), Université LavalQuébec, QC, Canada G1V 0A6; ‡ Department of Biology, Indiana UniversityBloomington IN, USA 47405; § Department of Biology, Saint Louis University, St. LouisMO 63103, USA; ¶ Sri Paramakalyani Centre for Environmental Sciences, Manonmaniam Sundaranar UniversityAlwarkurichi, Tamil Nadu, India; ** Department of Ecology and Biology of Fishes, Leibniz-Institute of Freshwater Ecology and Inland Fisheries12587 Berlin, Germany; †† Department of Zoology, University of DhakaDhaka, Bangladesh; ‡‡ Department of Zoology, Tribhuvan UniversityKathmandu, Nepal; §§ Wellcome Trust Sanger InstituteCambridge, UK; ¶¶ The Genome Analysis CentreNorwich, UK

**Keywords:** genetic subdivision, genomics, outlier analysis, single nucleotide polymorphisms, zebrafish

## Abstract

Understanding a wider range of genotype–phenotype associations can be achieved through ecological and evolutionary studies of traditional laboratory models. Here, we conducted the first large-scale geographic analysis of genetic variation within and among wild zebrafish (*Danio rerio*) populations occurring in Nepal, India, and Bangladesh, and we genetically compared wild populations to several commonly used lab strains. We examined genetic variation at 1832 polymorphic EST-based single nucleotide polymorphisms (SNPs) and the *cytb* mitochondrial gene in 13 wild populations and three lab strains. Natural populations were subdivided into three major mitochondrial DNA clades with an average among-clade sequence divergence of 5.8%. SNPs revealed five major evolutionarily and genetically distinct groups with an overall *F*_ST_ of 0.170 (95% CI 0.105–0.254). These genetic groups corresponded to discrete geographic regions and appear to reflect isolation in refugia during past climate cycles. We detected 71 significantly divergent outlier loci (3.4%) and nine loci (0.5%) with significantly low *F*_ST_ values. Valleys of reduced heterozygosity, consistent with selective sweeps, surrounded six of the 71 outliers (8.5%). The lab strains formed two additional groups that were genetically distinct from all wild populations. An additional subset of outlier loci was consistent with domestication selection within lab strains. Substantial genetic variation that exists in zebrafish as a whole is missing from lab strains that we analysed. A combination of laboratory and field studies that incorporates genetic variation from divergent wild populations along with the wealth of molecular information available for this model organism provides an opportunity to advance our understanding of genetic influences on phenotypic variation for a vertebrate species.

## Introduction

To link genotype to organismal phenotype, studies must integrate across levels of biological organization. These levels or organization include variation at the species level, the interaction of evolutionary process within and among populations, individual phenotypic variation, and gene activities underlying phenotypic variation (Dalziel *et al.* 2009). One research approach in this direction is to study natural populations of traditional laboratory models, for which a wide array of genomic resources and molecular genetic tools exist. These resources include whole-genome sequence, mutant phenotypes linked to genes in lab strains, and readily available panels of single nucleotide polymorphisms (SNPs) and other genetic markers (Stapley *et al.* 2010). The combination of genomic data and ecological information from natural populations of organisms such *Drosophila*, the mouse or *Arabidopsis* has made it possible to address fundamental questions in ecology and evolution such as unravelling complex gene networks underlying adaptive evolution (Steiner *et al.* 2007; Rebeiz *et al.* 2009; Brachi *et al.* 2010; Turner *et al.* 2011).

The zebrafish (*Danio rerio*) is a prominent model organism in developmental genetics, neurophysiology and biomedical research (Lieschke & Currie 2007; Spence *et al.* 2008). Currently, over 400 laboratories worldwide conduct research with zebrafish from established lab strains largely because of its short generation interval, rapid development, high fecundity, transparent embryos and ease of genetic manipulation (Lieschke & Currie 2007). As a result of this prominence, full-genome sequence is available for this species, a wide array of well-characterized mutant and transgenic phenotypes exist, and molecular genetic techniques such as targeted gene knockdown using morpholino antisense oligos are well established (Lieschke & Currie 2007; Kishi *et al.* 2009). Recent studies have begun to examine questions in ecology and evolution using natural populations of zebrafish, which occur in India, Nepal and Bangladesh (Engeszer *et al.* 2007; Spence *et al.* 2008). These include: behavioural genetics of shoaling, activity level, boldness and aggression (Moretz *et al.* 2007), feeding ecology (McClure *et al.* 2006), reproductive behaviour (Hutter *et al.* 2010), colour and pattern variation as it relates to speciation (Parichy 2006), genetic effects of domestication (Robison & Rowland 2005; Robison 2007), variation in individual growth rates (Spence *et al.* 2007) and the number of recessive lethals in wild-caught populations (McCune *et al.* 2002). However, much more potential exists to link extensive knowledge of development and phenotypic expression with genes and gene networks underlying ecologically important traits in this species.

An analysis of existing genetic diversity and historical evolutionary relationships both among natural populations and between natural populations and established lab strains is needed as a foundation for further ecology and evolution studies of zebrafish. Zebrafish occur over a wide geographic range (Spence *et al.* 2008), and there is a strong possibility that major phylogeographic breaks occur. In addition, past breeding practices and collection from limited natural populations may have lead to marked divergence between lab strains and wild populations. However, little is known about these evolutionary relationships (Engeszer *et al.* 2007; Coe *et al.* 2009). Previous work on wild zebrafish populations was performed using a small number of neutral markers and revealed low levels of population substructure for several geographically proximate natural populations in northeastern India (Gratton *et al.* 2004). Furthermore, several studies have examined genetic variation within and among lab strains (Guryev *et al.* 2006), including one that found that lab strains have reduced genetic variation compared with one wild population (Coe *et al.* 2009).

In this paper, our main objective was to provide the first population genomic analysis of wild zebrafish populations on a large geographic scale. More specifically, we tested the hypothesis of lineage diversification among natural populations and compare genetic diversity among wild populations and lab strains for the mitochondrial and nuclear genomes. To achieve this, we documented phylogeographic relationships and hierarchical population structure. Finally, we tested for the effects of selection in driving patterns of divergence and diversity at individual loci across the genome.

## Material and methods

### Samples

The current zebrafish species range is centred around the Ganges and Brahmaputra Rivers in northeastern India, low-lying Nepal and Bangladesh (Engeszer *et al.* 2007). Disjunct extant populations occur in southwestern India in the Western Ghats mountain range (Engeszer *et al.* 2007). There are records of zebrafish collections from central India from as recently as the 1970’s (Engeszer *et al.* 2007), but there is some taxonomic uncertainty regarding these records (Spence *et al.* 2008) and this species has not been observed in central Indian locations more recently.

**Fig. 1:**
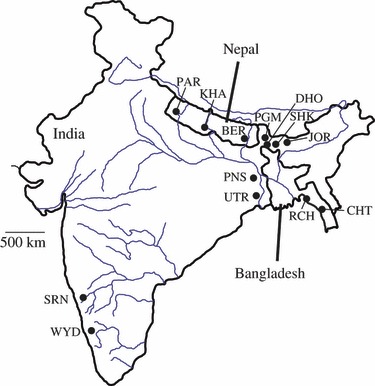
Map of study area (India, Nepal, Bangladesh) with sampling locations (black circles) and corresponding abbreviations from Table 1 for wild population samples.

We collected zebrafish from 13 wild populations from Nepal, India and Bangladesh and three common lab strains (AB, SJA, and TM1; Table 1, Fig. 1). Mean sample size was 15.3 and ranged from 2 to 20 (Table 1). Fish were collected directly from field locations with a combination of sampling techniques (seine, cast nets or dip nets). Either whole fish or fin clips were preserved in 95% ethanol until DNA extraction. All necessary collection permits were obtained.

**Table 1:** Sample locations, abbreviations, geographic locations and sample sizes for both mitochondrial DNA (mtDNA) (*N*_mtDNA_) and single nucleotide polymorphisms (SNPs) (*N*_SNP_). SHK could not be examined with SNPs, and mtDNA sequence was not examined for DHO. SRN and WYD were not used for most SNP analyses because of small sample sizes (see Results)

Location	Abbreviation (ID)	Latitude	Longitude	*N*_mtDNA_	*N*_SNP_
Paruwa Sota River, Western Nepal	PAR	28.125°	81.799°	15	19
Khair Khola, Central Nepal	KHA	27.618°	84.533°	15	19
Bering River, Eastern Nepal	BER	26.642°	87.937°	15	19
Shikarpur, near Coochibihar, West Bengal, India	SHK	26.321°	89.463°	10	—
Dharola, India	DHO	26.282°	89.237°	—	15
Jorai, India	JOR	26.497°	89.821°	15	17
Panigram, India	PGM	26.436°	89.163°	13	19
N. Parganas, India	PNS	22.879°	88.767°	15	20
Uttarbhag, India	UTR	22.361°	88.506°	15	19
Rice paddy between Dhaka and Chittagong, Bangladesh	RCH	23.518°	90.851°	14	14
Chittagong, Bangladesh	CHT	22.474°	91.783°	15	18
Sringeri, Thunga R., Karnataka, India	SRN	13.417°	75.251°	3	3
Wayanad, Karampuzha Dam, Kerala, India	WYD	11.619°	76.174°	2	2
AB lab strain	AB	—	—	10	15
SJA lab strain	SJA	—	—	10	15
TM1 lab strain	TM1	—	—	10	5

Zebrafish lab strains have generally been developed without consideration of wild origin. Approximately 18 ‘wild-type’ zebrafish lab strains have been established from a limited number of wild-caught individuals from several sampling events that occurred in geographically restricted locations (Spence *et al.* 2008). These lab strains have generally been bred for reduced genetic diversity and purging of lethal mutations as an aid to molecular biology research (Spence *et al.* 2008). We examined three lab strains in this study: AB, SJA and TM1. The AB line was developed with fish purchased from a U.S. pet store in the 1970’s (Spence *et al.* 2008). It has been maintained since then at the Zebrafish International Resource Center (ZIRC). SJA is an inbred line derived from AB (Spence *et al.* 2008). TM1 was independently derived from a pet store in 1986 and is now approximately 30 generations removed from that point (Robison & Rowland 2005). We obtained individuals from all three strains from the ZIRC. DNA was extracted from fin clips from wild-caught and lab strain fish with the Pure Gene® kit (Gentra Systems) following the manufacturer’s instructions.

### Mitochondrial DNA (mtDNA)

We amplified a 1122 bp region of the cytochrome b (*cytb*) gene with primers modified from Fang *et al.* (2009) and Mayden *et al.* (2007). We modified the primers Fishcytb-F from Fang *et al.* (2009) to create Fishcytbzf-F (5′-ACCACTGTTGTAGTTCAACTACAAGAAC-3′). We used HA-danio from Mayden *et al.* (2007) as the reverse primer. A forward internal primer (cytb397-F; 5′-TTCTGAGGGGCCACAGTAAT-3′) and a reverse internal primer (cytb620-R; 5′-GGGGTTATTTGATCCGGTTT-3′) were used to obtain full sequences in both directions. PCRs (25 μL) were composed of 1× PCR buffer, 2 mm MgCl2, 0.2 mm of each dNTP, 1 μm of each primer, 1.25 U *Taq* polymerase and approximately 100 ng DNA. The PCR profile was as follows: 94 °C for 2 min, 33 cycles of 94 °C for 30 s, 54 °C for 30 s, 72 °C for 2.5 min, 72 °C for an additional 5 min and 4 °C until manually terminated. Standard Sanger sequencing was performed on both strands of DNA for each individual. Sequences were aligned manually with CODONCODE ALIGNER ver. 3.0.2 (CodonCode Corporation). All sequences have been deposited at Genbank (Accession numbers JN234180–JN234356).

### mtDNA diversity within and among populations

Haplotype diversity (*h*), nucleotide diversity (*π*)(Nei 1987) and net nucleotide differences per site (*Da*) (Nei & Lin 1979) were estimated with ARLEQUIN ver. 3.5.1.2 (Excoffier & Lischer 2010). We performed a model selection analysis for base substitution between haplotypes with PAUP 4.0 beta (Swofford 2003) and MODELTEST ver. 3.7 (Posada & Crandall 1998). The selected model under both AICc and BIC was TrN + I (Tamura & Nei 1993), which was then used to correct genetic distances in subsequent analyses. We used pairwise *Φ*_ST_ based on the TrN substitution model to estimate genetic differentiation among populations (Excoffier *et al.* 1992). A total of 10 000 permutations were performed to estimate significance levels, which we then corrected for false discovery rate with α = 0.05 (Benjamini & Hochberg 1995). We tested for population structure and major genetic assemblages with samova ver. 1.0 (Dupanloup *et al.* 2002). We tested *K* = 2 through *K* = 15 and chose the *K* with the highest *F*_CT_ value (the proportion of total genetic variation partitioned among groups of populations), based on the uncorrected *p*-distances used by samova. Additional analyses for the structure indicted by the chosen *K*-value were performed with ARLEQUIN. For this amova, we used the TrN substitution model with 10 000 permutations.

Phylogenetic analyses of haplotypic variation were conducted with MrBayes ver. 3.1.2 (Ronquist & Huelsenbeck 2003). This software does not implement the TrN model, so the GTR + I + G model was used. This is a more complicated substitution model but this Bayesian approach does not usually show poor performance when fitting a more complicated model (Ronquist *et al.* 2005). We performed two runs each with five chains and sampled every 1000 steps until standard deviations between split frequencies were <0.01. The first 25% of trees were discarded. The closely related *Danio kyathit* (Mayden *et al.* 2007) was used as an outgroup.

### SNPs

Single nucleotide polymorphism genotypes were collected with a custom zebrafish Affymetrix SNP array following the manufacturer’s protocol at the Génome Québec Innovation Center, Montréal, Canada. This array contained a combination of confirmed and predicted SNPs in gene transcripts based on Stickney *et al.* (2002) and Guryev *et al.* (2006). Stickney *et al.* (2002) identified SNPs in previously mapped genes on the basis of polymorphisms between the zebrafish C32 and SJD strains. The Guryev *et al.* (2006) data set contains over 50 000 predicted SNPs obtained by the comparison of EST traces from the WashU Zebrafish EST project (Clark *et al.* 2001), normally of known-strain cDNA libraries (e.g. SJD, C32 and AB), to genomic sequence traces from the Sanger Zebrafish genome project (Tubingen strain). In our SNP selection, we prioritized experimentally confirmed SNPs over predicted SNPs (confirmed SNPs are those that are experimentally polymorphic in at least one comparison of the AB, C32, TL, Tu and WIK strains). We also required that SNPs could not overlap or be within 50 bp of an already placed SNP. Finally, we attempted to generate an even spread across the genome. The selection consists of four subsets, as follows: (1) confirmed SNPs from Guryev *et al.* (2006) (*N* = 190), (2) confirmed SNPs from Stickney *et al.* (2002) (*N* = 66), (3) high-quality predicted SNPs from Guryev *et al.* (2006)(each allele was confirmed by two sequencing reads; *N* = 1245) and (4) high-quality predicted SNP from Guryev *et al.* (2006), where one of the alleles was confirmed by only one sequencing read (*N* = 6837). We used all of subsets 1, 2 and 3 and sampled through subset 4 seeking to fill gaps and achieve an even distribution up to a total of 3212 SNPs. Based on the sequences we supplied, Affymetrix (Santa Clara, CA) developed a Custom Affymetrix Targeted Genotyping assay. This assay is based on the Molecular Inversion Probes (MIPs) approach (Hardenbol *et al.* 2003). Map locations for the vast majority of SNPs are known based on map locations from the Zv9 zebrafish genome assembly. All SNP genotypes have been deposited at DRYAD (doi:10.5061/dryad.505dp), and locus-specific SNP information is available at Genbank.

### SNP diversity within and among populations

We performed an initial filter of the SNP data set to remove fixed loci and loci with minimum allele frequencies (MAF) <1%. This resulted in 1832 variable SNPs among all populations and lab strains. We tested for Hardy–Weinberg proportions in each population or lab strain with GENEPOP ver. 4 (Raymond & Rousset 1995). GENETIX ver. 4.05 (Belkhir 1999) software was used to estimate *θ* analogues (Weir & Cockerham 1984) of *F*_ST_. We used the DEMEtics ver. 0.8-3 (Gerlach *et al.* 2010) package for R ver. 2.12 (R Development Core Team 2006) to estimate *D*_est_ (Jost 2008). One thousand permutations were performed to calculate 95% confidence intervals or *P*-values for both measures.

For the analysis of population groups across geographic space, we used STRUCTURE ver. 2.3.1 (Pritchard *et al.* 2000) to estimate the number of population clusters (*K*) with the highest log likelihood. For STRUCTURE analyses, we did not incorporate prior population information. We used 100 000 replicates and 20 000 burn-in cycles under an admixture model. We inferred a separate α for each population (α is the Dirichlet parameter for degree of admixture). We used the correlated allele frequencies model with an initial *λ* of 1, where *λ* parameterizes the allele frequency prior and is based on the Dirichlet distribution of allele frequencies. We allowed *F* to assume a different value for each population, which allows for different rates of drift among populations. We performed 10 runs for each of *K* = 1–15, the total number of population samples examined with SNPs. We performed two rounds of analysis with STRUCTURE. For the first round, in addition to the initial filter for fixed loci and MAF <1%, we also filtered the data set for linkage disequilibrium (LD). One locus in a pair was randomly removed from the data set if estimated *r*^2^ was >0.5. The LD filter resulted in a set of 522 SNPs distributed throughout the genome (mean number of SNPs per chromosome = 21.8, mean distance between markers = 2.9 Mb). After the first round of STRUCTURE analysis, we performed a hierarchical outlier locus analysis (Excoffier *et al.* 2009) to identify loci putatively influenced by natural selection (see next section). We filtered putatively selected loci from the data set prior to a second round of STRUCTURE analysis.

Estimates of genetic diversity and divergence with SNPs like those used in this study, which were developed from lab strains, may be prone to ascertainment bias. Ascertainment bias did not appear to have a large influence on allele frequency spectra for the wild populations but did appear to have an influence on results for lab strains (see Supporting information). We therefore performed analyses with and without the lab strains.

### SNP hierarchical outlier locus analysis

We conducted an outlier analysis with the hierarchical FDIST model (Beaumont & Balding 2004; Excoffier *et al.* 2009) implemented with ARLEQUIN ver. 3.5.1.2. We used this approach because of the regional genetic structure present in our data. Excoffier *et al.* (2009) demonstrated an increased false positive rate when hierarchical genetic structure is present but not accounted for in outlier locus analyses. Further, we could not have examined all of our population samples collectively without violation of the assumption of sample exchangeability during the ‘scattering’ phase of the models implemented in BAYESFST (Beaumont & Balding 2004) or BAYESCAN (Foll & Gaggiotti 2008). For the hierarchical FDIST model, 30 000 simulations were conducted with 20 simulated groups each with 100 demes. We applied a significance cutoff of *P* < 0.01. To reduce the number of potential false positives, we reported only loci with scaled heterozygosities [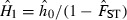] >0.2, following Excoffier *et al.* (2009). We used the AmiGO browser of gene ontology (http://www.geneontology.org), the KEGG PATHWAY database (http://www.genome.jp/kegg/pathway.html) and the UniProt database (http://www.uniprot.org/), along with corresponding literature searches to assign significant outlier SNPs to putative functional groups.

We conducted the hierarchical FDIST outlier analyses with the larger 1832 SNP data set (filtered only for fixed loci and loci with MAF <1%). We did not apply the LD filter for this analysis so that all potential candidate loci and regions of chromosomes had the opportunity to be detected. We excluded lab strains from the analyses initially to estimate outliers among natural populations (excluding the two sites in southern India, SRN and WYD, because of small sample size). We used the population structure consistent with the *K* = 7 STRUCTURE model (see SNP divergence among populations in Results section below) to account for substructure within the hierarchical FDIST model. The five groups of natural populations were: PAR, KHA and CHIT each formed a separate group; BER, DHO, JOR, PGM and RCH formed a group; and PNS and UTR formed the final group. We chose to split the latter two populations from the other sites in this genetic group because over-splitting is likely to have less of an effect on false positive rates for this analysis than under-splitting (Excoffier *et al.* 2009).

We conducted a second hierarchical outlier locus analysis with individuals from all wild populations (again excluding SRN and WYD from southern India) and including the three lab strains. We used the population structure consistent with the *K* = 7 STRUCTURE model. Groups were defined as follows: PAR, KHA and CHIT each formed a separate group; BER, DHO, JOR, PGM and RCH formed a group; PNS, UTR and TM1 formed a group; and AB and SJA were grouped together. The inclusion of AB and SJA as one or as two groups did not influence results (data not shown).

Nonrandom aggregations of significant *F*_ST_ outliers along chromosomes would be consistent with ‘hotspots’ or genomic regions with multiple loci that have been influenced by selection. To test for nonrandom outlier aggregations, we divided the genome into 20 Mb bins. This resulted in a total of 76 bins among the 25 zebrafish chromosomes. We assumed a Poisson distribution to calculate the probability of observing a given number of significant high or low *F*_ST_ outliers within a bin, following Brem *et al.* (2002). The mean of the Poisson distribution for each data set was estimated as 76 bins divided by the number of significant outliers at the cutoff of *P* < 0.01, and we performed calculations separately for high and low outliers. For the analysis that excluded lab strains (natural populations only), the probability of observing four significant high outliers in a given window was 0.008. The probability of observing three significant high outliers was 0.04. The probability of observing two significant low outliers was 0.006. For the analysis that included wild populations and lab strains, the probability of observing four significant high outliers in a given 20 Mb bin was 0.015, and the probability of observing five high outliers was 0.003. The probability of observing two significant low outliers was 0.036.

Selection is expected to influence the levels of genetic diversity in the vicinity of target loci. If selection is responsible for the elevated *F*_ST_ values of outlier loci, linked variation should be ‘swept’ through the population along with the advantageous locus (Akey 2009; Hernandez *et al.* 2011). Short-term balancing selection might also lead to reduced genetic diversity in the vicinity of the selected locus; however, in the longer-term balancing selection may lead to increased diversity close to selected sites (Charlesworth 2006). To test for reduced heterozygosity surrounding outlier loci, we calculated heterozygosity within 10 Mb windows surrounding each significant outlier locus and compared this to a genome-wide distribution of heterozygosity within nonoutlier windows. We used nonoverlapping 10 Mb sliding windows from throughout the genome to calculate the genome-wide distribution from nonoutlier windows. This window size was chosen because it contained an adequate number of SNPs within windows (mean number of SNPs within windows = 14.5). Mean heterozygosity was calculated within a window if at least six SNPs were present. Windows were constrained to occur outside of 10 Mb windows surrounding each outlier locus. The percentile at which each outlier locus occurred was used as a *P*-value for deviation from the genome-wide average and a significance cutoff of *P* < 0.05 was used.

We performed tests for reduced heterozygosity surrounding outlier loci separately for a representative wild population and a representative lab strain. For the representative wild population, we pooled individuals from BER, DHO, JOR, PGM to increase power (pooled *N* = 68). These populations had very low levels of genetic differentiation (mean pairwise *D*_est_ = 0.005, mean pairwise *F*_ST_ = 0.019) and belonged to the same genetic cluster in STRUCTURE models (see Results below). We used the AB strain as the representative lab strain. The sample size for AB was larger than TM1 (Table 1). The AB strain should be more representative of other lab strains than SJA because SJA has been bred to reduce genetic variation as much as possible (Spence *et al.* 2008). For the wild population test, we used outliers from the hierarchical analysis that excluded lab strains. For the lab strain test, we used outliers from the hierarchical analysis that included lab strains.

Linkage disequilibrium may also be elevated in regions surrounding outlier loci. We estimated the parameter *r*^2^ with the software PLINK ver. 1.07 (Purcell *et al.* 2007) as a measure of LD that is less biased by rare alleles than other measures (Eberle *et al.* 2006; VanLiere & Rosenberg 2008). We first determined overall patterns and extent of LD within the genomic background of wild and lab strain populations. We calculated the half-length of *r*^2^, that is, the distance in bp at which *r*^2^ reach 50% of its maximal value, and the distance at which *r*^2^ reached 0.2. We performed this analysis separately for the same representative wild population and the AB lab strain. In both cases, we binned syntenic SNP pairs in 5 Mb intervals for each chromosome, calculated mean *r*^2^ within the intervals, and fitted a logarithmic curve to the data (Gray *et al.* 2009).

To test for elevated LD surrounding outlier loci, we estimated *r*^2^ within 10 Mb windows surrounding each significant outlier locus and compared this to a genome-wide distribution of LD values in nonoutlier windows. Nonoutlier windows were constrained to occur outside of 10 Mb windows surrounding each outlier locus. We estimated *r*^2^ between each of the surrounding SNPs and the focal outlier or central locus within a nonoutlier window. Mean LD within nonoutlier windows was used to create a genome-wide distribution and the percentile at which mean LD for each outlier region deviated from the genome-wide average was used as a *P*-value. We used a significance cutoff of *P* < 0.05. We performed these tests for the same representative wild population and representative lab strain as the tests for reduced heterozygosity near outliers.

## Results

### mtDNA diversity within populations

Sequence data for the 1122 bp region of the *cytochrome* b (*cytb)* gene examined in 177 zebrafish revealed a total of 67 haplotypes defined by 174 segregating sites of which 156 were parsimony informative. There were no gaps in the alignment. Transitions were observed 22 times more often than transversions, as estimated with MrBayes. Base composition was biased towards A and T nucleotides (61% AT content). A low estimate of the shape parameter of the gamma distribution (α = 0.097) indicated pronounced heterogeneity of substitution rate over sites.

The number of haplotypes per population ranged from 1 to 10 (Table 2). The mean number of segregating sites within populations was 18.0 (range 0–67). Mean haplotypic diversity (*h*) was 0.64 (range 0–0.92), and mean nucleotide diversity (*π*) was 0.45% (range 0–1.3%). RCH (in Bangladesh) had an extreme number of segregating sites (*S* = 67). Haplotypic diversity was not the greatest in this population, but nucleotide diversity was (Table 2). In contrast, each of the lab strains (AB, SJA and TM1) was monomorphic (Table 2).

**Table 2:** Genetic diversity summary statistics for zebrafish from India, Nepal, Bangladesh and three lab strains

	mtDNA				SNPs
	
ID	*S*	*h* (SD)	*π*% (SD)	Haplotypes observed	*H*_S_
PAR	25	0.87 (0.05)	0.88 (0.48)	2–8	0.154
KHA	13	0.92 (0.05)	0.24 (0.15)	49–57	0.060
BER	24	0.90 (0.07)	0.40 (0.23)	20, 40–48	0.223
SHK	16	0.89 (0.08)	0.53 (0.31)	16, 17, 20, 30, 58, 59	—
DHO	—	—	—	—	0.226
JOR	13	0.64 (0.13)	0.26 (0.16)	16, 17, 18, 19, 20	0.224
PGM	17	0.92 (0.05)	0.55 (0.31)	16, 17, 20, 21, 22, 23, 24, 25	0.253
PNS	20	0.83 (0.08)	0.55 (0.31)	20, 26, 27, 28, 29, 30, 31	0.272
UTR	20	0.90 (0.05)	0.62 (0.35)	9, 20, 26, 61, 62, 63, 64, 65	0.219
RCH	67	0.89 (0.06)	1.30 (0.70)	32–39	0.215
CHT	19	0.57 (0.15)	0.49 (0.28)	10–15	0.068
SRN	0	—	—	60	—
WYD	1	—	—	66, 67	—
AB	0	0	0	9	0.142
SJA	0	0	0	9	0.027
TM1	0	0	0	20	0.235

**Fig. 2:**
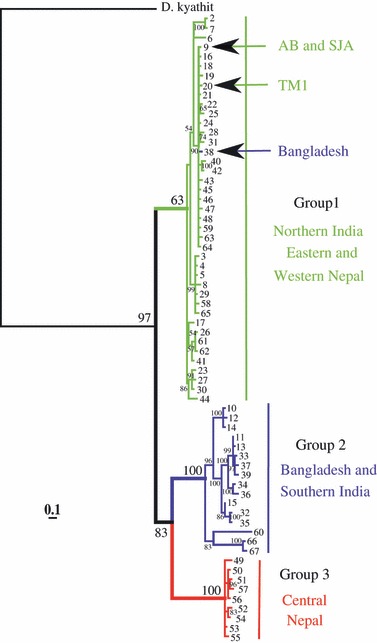
Bayesian mitochondrial DNA (mtDNA) phylogenetic analysis of zebrafish haplotypes from wild populations and lab strains. Numbers at branch tips are haplotypes referred to in Table 1. Three genetic groups were defined by mitochondrial DNA (mtDNA) (perpendicular lines) and labelled according to sampling locations. Haplotypes are colour coded according to these groups. The scale shows mean expected number of substitutions per site. Numbers along branches show posterior probabilities of nodes.

Mitochondrial DNA (mtDNA) diversity is represented by *S*, number of segregating sites, *h*, haplotype diversity and π, nucleotide diversity. Standard deviations are in parentheses. Numbers assigned to haplotypes in the ‘haplotypes observed’ column correspond to Fig. 2. SNP diversity is summarized by *H*_S_, mean unbiased expected heterozygosity within populations or lab strains. SHK was not examined with single nucleotide polymorphisms (SNPs), and mtDNA sequence was not examined for DHO. Genetic diversity summary statistics are not presented for samples SRN and WYD because of small sample size.

### mtDNA divergence among populations

Mitochondrial genetic structure corresponded to geographic regions. Estimates of *Φ*_ST_ based on the TrN substitution model ranged from −0.05 to 1.0, and estimates of net nucleotide differences per site (*Da*) ranged from −0.03% to 7.06% (Table S1, Supporting information). Among populations near or adjacent to the Ganges and Brahmaputra Rivers in India and Nepal (BER, SHK, JOR, PGM, PNS and UTR), genetic differentiation tended to be low and nonsignificant, although several of the pairwise comparisons that included BER and UTR were significant (Table S1, Supporting information). These populations also commonly shared haplotypes (Table S1, Supporting information). The samples from western Nepal (PAR), central Nepal (KHA), Bangladesh (RCH and CHT) and southern India (SRN, WYD) were each genetically differentiated from other sites (Table S1, Supporting information). The lab strains AB and SJA were fixed for the same haplotype, and the strain TM1 was fixed for a genetically similar haplotype. These three lab strains were highly genetically similar to the populations from northern India and eastern Nepal (Table S1, Supporting information).

samova analysis supported these interpretations of phylogeographic structure. *K* = 6 had the greatest support, that is, it had the highest among-group variance component (*F*_CT_ = 88.3, *P* < 0.001). KHA, RCH, CHT, SRN and WYD each formed its own group. PAR, BER, SHK, JOR, PGM, PNS, UTR, AB, SJA and TM1 all fell within an additional group. Additional calculation of an amova with 10 000 permutations and genetic distances based on the TrN model yielded a corrected *Φ*_CT_ of 88.8 (*P* = 0.0005). Overall *Φ*_ST_ was 0.91 (*P* < 0.0001), and variation within groups (*Φ*_SC_) was 0.20 (*P* < 0.0001). That latter variance component reflects variation among populations within the group that contained 10 populations, as all other groups each only contained one population.

**Fig. 3:**
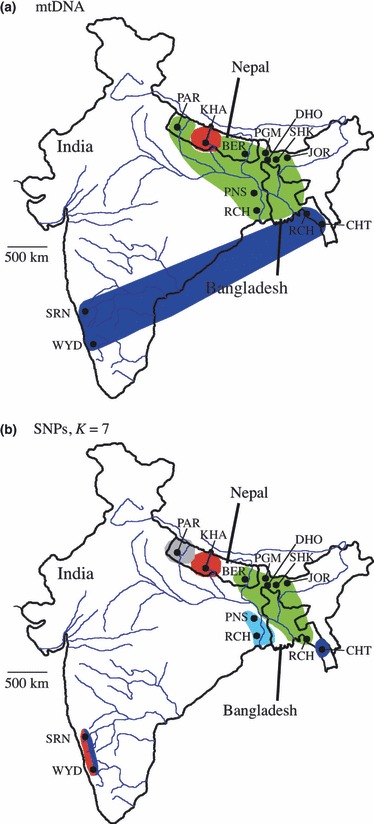
Map of India with sampling locations (black circles), sample abbreviations and colour-coded genetic clusters of wild populations from (a) mitochondrial DNA (mtDNA) analysis and (b) single nucleotide polymorphism (SNP) analysis with STRUCTURE.

Bayesian phylogenetic analyses of the 67 haplotypes revealed three major genetic groups corresponding to Northern India and western and eastern Nepal (Group 1, green; Figs 2 and 3a), Bangladesh/southern India (Group 2, blue; Figs 2 and 3a) and Central Nepal (Group 3, red; Figs 2 and 3a). Subdivision into these three assemblages was well supported, as indicated by nodal posterior probabilities (Fig. 2), and represents deep historical evolutionary divergence. Mean percentage nucleotide differences among the groups were: 5.4% (Group 1–2), 5.6% (Group 1–3) and 6.3% (Group 2–3). The three lab strains belonged to the northern India genetic assemblage (Group 1; Fig. 2). One haplotype from one individual sampled in RCH (Bangladesh) belonged to Group 1 and likely corresponds to an individual with migrant ancestry (Fig. 2).

### SNP diversity within populations

With the 1832 SNP data set, a total of 11 593 tests for H-W proportions were possible. We observed 639 significant results for tests of deviation from H-W proportions, close to that expected because of chance alone (expected 580 significant tests at α = 0.05). Mean expected heterozygosity for SNPs was not significantly lower for the three lab strains together (mean = 0.134, *SD* = 0.104) compared with wild populations (mean = 0.191, SD = 0.073; *t* = 1.08, d.f. = 11, *P* = 0.15). However, SJA clearly showed highly reduced diversity relative to the other two lab strains (Table 2) and AB had substantially reduced genetic diversity (mean *H*_e_ = 0.142) relative to wild populations collected near the Ganges and Brahmaputra Rivers in northern India and Nepal (range of mean *H*_e_: 0.223–0.253).

### SNP divergence among populations

We removed significant outlier loci from the data set prior to analysis of genetic population differentiation (see Outlier locus analysis below). A data set of 479 SNPs remained following filtration for fixed loci, MAF <1%, LD and outliers. Overall *F*_ST_ with lab strains included was 0.234 (95% CI 0.229–0.239) and overall *D*_est_ was 0.104 (95% CI 0.103–0.106). With lab strains removed, overall *F*_ST_ was 0.170 (95% CI 0.105–0.254) and overall *D*_est_ was 0.085 (95% CI 0.084–0.086). All pairwise *F*_ST_ and *D*_est_ estimates were significant after controlling the FDR (α = 0.05), except for the estimates between PGM and JOR for both measures (Table S2, Supporting information). Pairwise estimates of genetic differentiation were low between populations in northeastern India (DHO, JOR, PGM, PNS, UTR and RCH) and eastern Nepal (BER). Pairwise estimates of differentiation that included the central (KHA) and western (PAR) Nepal samples were greater. The southern Bangladesh population (CHT) was also genetically differentiated (Table S2, Supporting information). Each lab strain was highly differentiated from the wild populations.

**Fig. 4:**
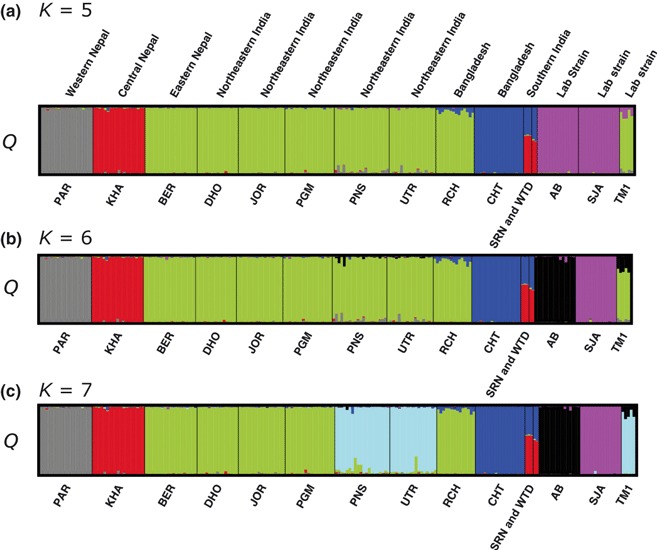
Proportion of the genome (*Q*) of each individual assigned by STRUCTURE to each population sample based on single nucleotide polymorphism (SNP) genotypes. Results correspond to models with (a) *K* = 5, (b) *K* = 6 and (c) *K* = 7. Each column corresponds to an individual, and sample locations are separated by vertical bars. Each of the seven clusters was given a separate colour that corresponds to Fig. 3b.

Patterns of genetic subdivision revealed by the STRUCTURE analysis of SNP data were generally consistent with the groups defined by the mitochondrial analysis but provided evidence for further genetic subdivision (Figs 3 and 4). For the analysis that included lab strains, estimated STRUCTURE model log-likelihoods increased from *K* = 1 to *K* = 7, after which estimated log-likelihoods reached an asymptote and variance among the 10 runs increased markedly (Fig. S3, Supporting information). The model with *K* = 5 revealed genetic differentiation between two Nepal sites (PAR and KHA), a group of northeastern India sites near the Ganges and Brahmaputra Rivers (Ganges/Brahmaputra group: BER, DHO, JOR, PGM, PNS, UTR, and RCH), the southern Bangladesh site (CHT) and the lab strains AB and SJA. The TM1 lab strain fell within the Ganges/Brahmaputra group. The southern India populations appeared to have split ancestry between KHA and CHT (Fig. 4a). The RCH sample, from Bangladesh, clustered with the northern India SNP group, instead of with CHT as it did for the mtDNA data (Fig. 2). The *K* = 6 STRUCTURE model revealed genetic differentiation between the AB and SJA lab strains, otherwise the groupings were the same as *K* = 5 (Fig. 4b). The *K* = 7 model revealed differentiation between the two Indian samples southwest of the Ganges River (PNS and UTR) from the samples north or east of the Ganges River (BER, DHO, JOR, PGM, and RCH; Fig. 4c). The TM1 lab strain clustered with the PNS/UTR group (Fig 4c). An additional STRUCTURE analysis that excluded lab strains excluded did not change inference of wild population groupings (data not shown).

### Outlier locus analysis

**Fig. 5:**
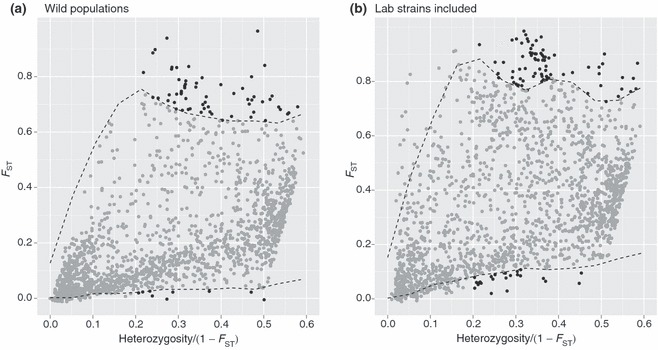
Hierarchical outlier locus analysis of (a) wild populations without lab strains and (b) wild populations with lab strains included. Black dotted lines show the 1% and 99% quantiles. Black filled circles represent loci significant at *P* ≤ 0.01 and with scaled heterozygosity >0.20. Heterozygosity on the *x*-axis is scaled by (1−*F*_ST_).

**Fig. 6:**
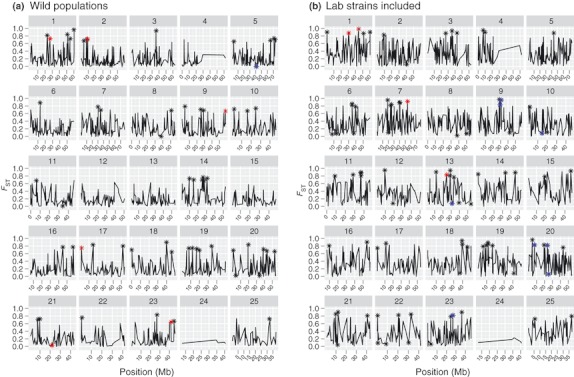
*F*_ST_ as a function of chromosome position for the outlier locus analysis that included (a) wild populations without lab strains and (b) wild populations with lab strains. Asterisks are shown for significant (*P* ≤ 0.01) high and low outlier loci. Red asterisks show outliers surrounded by a window of significantly reduced heterozygosity. Blue asterisks represent outliers surrounded by a window of significantly elevated LD.

Hierarchical outlier analysis of wild populations (excluding the lab strains) revealed 71 loci (*P* ≤ 0.01) with extreme genetic differentiation consistent with either directional (*N* = 62) or balancing (*N* = 9) selection (Figs 5a and 6a). The 62 significant high *F*_ST_ outliers occurred on 22 chromosomes (Fig. 6a). Clusters of outliers that significantly deviated from random expectations occurred on several chromosomes. Four high outliers (*P* = 0.008) occurred within a 20 Mb region on chromosome 14 and three high outliers (*P* = 0.04) occurred within 20 Mb regions on chromosomes 1, 2, 5 and 19. The nine significant low *F*_ST_ outliers were distributed on seven chromosomes (Fig. 6a). Seven of the 62 high outliers (11%) were nonsynonymous amino acid substitutions, five of which occurred in unknown genes (Table S3, Supporting information). One nonsynonymous substitution occurred in a gene putatively associated with metabolic processes (glucose-fructose oxidoreductase activity) and another with signal transduction (Table S3, Supporting information). The remaining significant high outliers were synonymous substitutions associated with various functions (Table S3, Supporting information). The only nonsynonymous low outlier (of nine) occurred in *influenza virus NS1A binding protein a*. The remaining low outliers were synonymous substitutions (Table S3, Supporting information).

The hierarchical outlier analysis that included lab strains revealed 99 loci (*P* ≤ 0.01) with extreme genetic differentiation consistent with either directional (*N* = 75) or balancing (*N* = 24) selection (Figs 5b and 6b). The 75 significant high *F*_ST_ outliers were distributed throughout the genome on 23 linkage groups. Four high outliers (*P* = 0.015) occurred within 20 Mb regions on chromosomes 11 and 13 (Table S4, Supporting information; Fig. 6b). Five high outliers (*P* = 0.003) within a 20 Mb window occurred on chromosome 19 (Table S4, Supporting information; Fig. 6b). The 24 significant low *F*_ST_ outliers were distributed on 14 chromosomes. Two occurred within a 20 Mb region (*P* = 0.036) on chromosomes 6, 13 and 22. Sixteen of the 75 (21%) high outliers and four of the 22 (18%) low outliers were also identified when lab strains were excluded from the analysis. The rank correlation between *P-*values for significant outliers with and without the lab strains was not significant (Spearman’s *ρ* = 0.123, *P* = 0.603).

The remaining outliers (59 high outliers and 20 low outliers) were significant only when lab strains were included in the analysis and are therefore candidates for the influence of domestication selection. Seven of these 59 high outliers (12%) were nonsynonymous substitutions. These genes were putatively associated with oxidoreductase activity, metabolic processes, chromatin assembly/disassembly and an intermediate filament associated protein of unknown function (Table S4, Supporting information). The remaining 52 of these high outliers were synonymous substitutions with various functions, one of which was muscle contraction (*tropomyosin* 3; Table S4, Supporting information). The subset of 20 low outliers unique to the analysis with the inclusions of lab strains contained one nonsynonymous substitution (putatively association = metabolic processes), the remainder were synonymous substitutions (Table S4, Supporting information).

### Hitchhiking surrounding outlier loci

For the wild population, heterozygosity was significantly reduced near six of 71 outlier loci (8.5%) compared with genome-wide average heterozygosity in 10 Mb windows (*P* < 0.05; Table S3, Supporting information; Fig. 6a). Windows with significantly reduced heterozygosity occurred throughout the genome (Fig. 6a). Five of these six loci were divergent outliers. Four of the five high outlier loci were associated with unknown genes (Table S3, Supporting information). One divergent outlier occurred in a putative transcription regulator (*paraspeckle component I*; Table S3, Supporting information). The low outlier with significantly reduced heterozygosity was associated with the *baculoviral IAP repeat-containing 2* gene, putatively involved with the regulation of apoptosis (Table S4, Supporting information). For the AB lab strain, heterozygosity was significantly reduced near four high outlier loci (*P* < 0.05; Table S4, Supporting information; Fig. 6b). Two of these genomic windows with significantly reduced heterozygosity occurred on chromosome 1 (unknown genes; Table S4, Supporting information). Two regions of reduced variation were associated with transcription factors: *transcription factor 12* (chromosome 7) and *TATA-box-binding protein* (chromosome 13; Table S4, Supporting information).

**Fig. 7:**
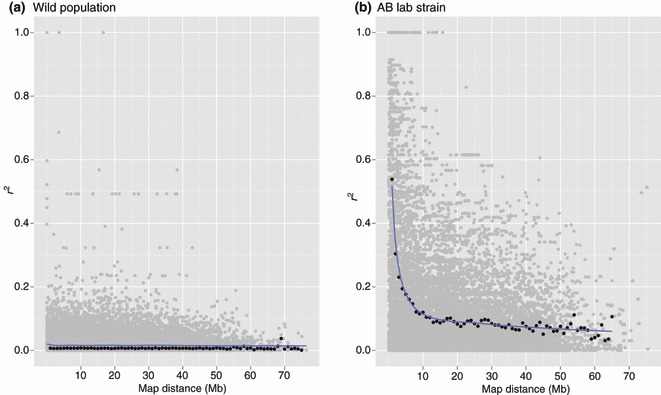
Decay plots of LD (*r*^2^) estimates for (a) a representative wild population (pooled individuals from BER, DHO, JOR and PGM) and (b) a representative lab strain (AB). Grey circles are pairwise *r*^2^. Black circles are the average *r*^2^ for each 1 Mb distance group for which a logarithmic trend line was fitted (solid lines; (a) *y* = −0.00082Ln (*x*) + 0.018, (b) *y* = −0.065Ln (*x*) + 0.306). In (b), mean *r*^2^ values were truncated at 65 Mb because of bias introduced by small number of data points in each 1 Mb interval beyond this point.

Overall levels of LD differed dramatically for wild populations and lab strains (Fig. 7). For the representative wild population, we tested LD among 1387 variable loci. Mean *r*^2^ was 0.016 (SD 0.029). The LD decay curve was generally flat (Fig. 7). The distance to reach either 50% of maximal *r*^2^ (genome half-length) or an *r*^2^ of 0.2 was <1 kb. The pattern and extent of LD were markedly different in the AB lab strain. Mean *r*^2^ was 0.135 (*SD* 0.201; *N* = 996 variable loci). LD decreased with distance in the AB strain (Fig. 7). The genome half-length was approximately 50 kb and the distance to reach an *r*^2^ of 0.2 was approximately 5.2 Mb.

Linkage disequilibrium was not significantly elevated surrounding any divergent outlier loci in the representative natural population but was significantly elevated surrounding one low outlier (*progestin and adipoQ receptor family member IIIa*; Table S3, Supporting information; Fig 6a). LD was significantly elevated surrounding five high and three low outliers in the AB lab strain (Table S4, Supporting information; Fig. 6b). The high outliers with elevated LD included *Kruppel-like factor 7* (chromosome 9; putative function = nucleic acid binding), an uncharacterized protein putatively involved with cell signalling (chromosome 20), and three unknown genes (chromosomes 9, 20 and 23; Table S3, Supporting information). The low outliers with elevated LD included *Sigma 1-type opioid receptor* (chromosome 10; putative function = transport), *DBH-like monooxygenase protein 1 homologue* (chromosome 20; putative function = metabolic processes) and an unknown gene (chromosome 13; Table S4, Supporting information).

## Discussion

### Divergence among wild populations and phylogeography

Our results revealed a central Ganges/Brahmaputra genetic group that contained subtle genetic substructure. This central group was surrounded by populations that exhibited deep phylogeographic divergence. Low genetic divergence among geographically proximate populations within the Ganges/Brahmaputra group is consistent with a previous genetic analysis of zebrafish from this region (Gratton *et al.* 2004) and is likely due to a combination of factors that lead to high gene flow (small differences in elevation among sites, large-scale flooding during the monsoon season and human-made irrigation channels and canals) and reduced effects of drift because of large subpopulation sizes. Substructure within this group occurred on either side of the Ganges and Brahmaputra Rivers and may be due to isolation by distance or an effect of these rivers themselves on dispersal.

Deep phylogeographic divergence of populations surrounding the central Ganges/Brahmaputra group is likely due to historical refugial effects during Quaternary climatic cycles. mtDNA sequence divergence allows approximate calculations of divergence times among several of the genetic groups. Based on a conventional molecular clock for *cytb* in fishes of 2% sequence divergence per million years (Bowen *et al.* 2001), levels of divergence were tentatively consistent with separation 2–3 million years ago. A number of opportunities for zebrafish population separation and isolation occurred during multiple dry and wet cycles during the Quaternary period in this region of Asia (Karanth 2003). Drier, colder and more seasonal periods were associated with glaciation events and weakening of the Asian southwest monsoon winds (Gupta *et al.* 2003). Increased desertification and reduction of tropical forests into savannah or patchy deciduous forests occurred during these periods (Brandon-Jones 1996; Meijaard & van der Zon 2003; Iyengar *et al.* 2005). Indeed, much of northern and western India may have been desert during glacial maxima (Fleischer *et al.* 2001) and tropical forests were replaced with savannahs in the foothills of the Himalayas (Karanth 2003).

These historical factors appear to have led to varying degrees of divergence of the zebrafish populations from the central Ganges/Brahmaputra genetic group. The populations from southern Bangladesh (CHT) and western (PAR) and central Nepal (KHA) likely occurred in separate Quaternary refugia. PAR was not as highly divergent from the Ganges/Brahmaputra group as the KHA population in terms of *cytb* sequence, but both PAR and KHA formed separate groups in the SNP STRUCTURE analysis. Differences in isolation time or secondary contact could explain these discrepancies. The RCH sample belonged to the CHT phylogeographic group based on mtDNA (except for one putative migrant from the Ganges/Brahmaputra group) and the Ganges Brahmaputra group based on SNPs. These results suggest that several historical or contemporary migration events have occurred between these sites. The two sites from southern India (SRN and WYD) belonged to the same mtDNA clade as CHT but exhibited mixed ancestry from central Nepal (KHA) and Bangladesh (CHT) based on SNPs. The SNP results are consistent with both the so-called Satpura hypothesis, which contends that fish moved from northern India through central India to reach the Western Ghats in southwestern India (Hora 1937; Silas 1952) and an alternative hypothesis that proposes that fish moved through riverine habitats within a previous land connection between southeast Asia and southern India (through the extant Indian Ocean; Daniels 2001). Separate waves of zebrafish arrival via each of the proposed routes would explain the SNP results, followed by retention of only haplotypes closely related to the CHT mtDNA clade.

### Lab strains

The three lab strains we examined were derived from U.S. pet stores, and the mtDNA results suggest that they originally were collected from the Ganges/Brahmaputra region, likely near the major city Kolkata (Calcutta). Fixation for separate mtDNA haplotypes between TM1 and AB/SJA is consistent with pet store lines that were obtained separately from the wild. Alternatively, a single sample from the wild could have contained several haplotypes that have subsequently become partitioned among pet store lines. The SNP STRUCTURE results were consistent with collection of the TM1 strain from the Kolkata region. If the AB and SJA lab strains were collected from the same region, as the mtDNA results suggest, the SNP results reveal that nuclear genomes have diverged since being brought into captivity. Ascertainment bias did not influence our interpretations for the loss of genetic diversity in lab strains based on mtDNA. The relative comparison of genetic divergence among wild populations and lab strains with this set of SNPs is likely robust to ascertainment bias, but further comparison of absolute values of divergence and diversity between lab strains and wild populations based on the SNP data would likely be biased. The observed pattern of reduced SNP diversity in both SJA and AB relative to Ganges/Brahmaputra wild populations is likely to be more pronounced with genomic markers not ascertained from lab strains.

Our results indicate that inbreeding and small effective population size (*N*_e_) of zebrafish lab strains has led to the predicted effects of reduced variation within and divergence among strains. Our scope of inference is limited to these three lab strains, but ‘wild-type’ lab strains for this species are generally derived from pet stores (although exceptions such as the strains Nadia and Darjeeling occur) followed by intentional inbreeding to remove lethal genes and to reduce genetic variation (Spence *et al.* 2008). Therefore, it is likely that advantageous alleles have been lost and deleterious alleles have generally been fixed in zebrafish lab strains. In lab strains of other model organisms, divergence among populations is responsible for variation in expressivity, penetrance and the effects of modifier loci among the genetic backgrounds of different lab strains (Nadeau 2001; Johnson *et al.* 2006). Differences in genetic background are likely responsible for strain-specific differences in zebrafish such as susceptibility to alcohol (Lockwood *et al.* 2004; Lieschke & Currie 2007) or selenium exposure (Benner *et al.* 2010).

Our study reveals that substantial genetic variation that exists in the zebrafish as a whole is missing from the three lab strains we examined. Therefore, associations between genotype and phenotype observed in these, and likely other, zebrafish lab strains could differ markedly within the genomic background of outbred wild populations. Inbreeding in lab strains generally causes reduced phenotypic variation (Coyne & Beecham 1987; Fowler & Whitlock 1999) and changes the additive genetic variance of traits (Falconer & Mackay 1996; Dworkin *et al.* 2005). In addition, recent work with *Drosophila* indicates that genotype-phenotype associations observed in the lab for traits such as bristle number (Gruber *et al.* 2007), mating discrimination behaviours (Barnwell & Noor 2008), wing shape (Dworkin *et al.* 2005) and viral resistance (Wilfert & Jiggins 2010) may not translate to wild populations.

### Evidence for selection

Selection appears to have influenced a small proportion of the genome in natural zebrafish populations. We observed significant outliers at 3.9% (71 of 1832) of the loci in our analysis of natural populations of zebrafish (lab strains excluded), which is a lower proportion than observed in several other EST-based SNPs studies to date (range 5.5–7.9%; Namroud *et al.* 2008; Narum *et al.* 2010; Renaut *et al.* 2011).

Of the 71 outlier loci from natural populations, multiple lines of evidence can be used to identify those that are most likely to have been influenced by selection. First, outliers associated with nonsynonymous substitutions may be the direct targets of selection. For example, the nonsynonymous divergent outlier SNP associated with glucose-fructose oxidoreductase activity may be associated with locally adapted metabolic differences among wild populations. The nonsynonymous low outlier SNP associated with the *influenza virus NS1A binding protein* represents a putative immune function-related gene (Wolff *et al.* 1998) under balancing selection among wild populations. Second, divergent outlier clusters may represent locally co-adapted gene complexes. The significant cluster on chromosome 14 also contained a nonsynonymous divergent outlier (*zgc:158426*, putatively associated with signal transduction function) and therefore represents an example of both of these lines of evidence. Third, a selective sweep is expected to cause a valley of reduced genetic variation and elevated LD around the target of selection (Charlesworth *et al.* 2003; Pennings & Hermisson 2006; Hernandez *et al.* 2011) and therefore would provide complementary information about locus-specific selective responses. The small number of outliers in the wild population with either significantly reduced heterozygosity or elevated LD in surrounding chromosomal regions is generally consistent with a lack of strong selective sweeps. This result could also be due to insufficient genomic resolution in our analysis or may be due to a history of soft sweeps that tend to leave small genomic footprints near selected loci, especially in the presence of gene flow (Pennings & Hermisson 2006; Allendorf *et al.* 2010). Those outliers that did exhibit reduced heterozygosity did so in the absence of elevated LD, which is consistent with a hard selective sweep in the not-too-distant but also not-too-recent past (Sabeti *et al.* 2006; Hohenlohe *et al.* 2010). Several outliers exhibited this pattern, including four divergent outlier loci. A candidate for balancing selection (*baculoviral IAP repeat-containing 2*) also showed this pattern, which suggests that depressed genetic variation surrounding a locus under balancing selection can persist long enough for LD to decay.

The outlier analysis that included lab strains revealed a subset of candidate genes or genomic regions for the influence of domestication selection. Divergent outliers with nonsynonymous substitutions had putative functions associated with metabolic processes, oxidoreductase activity and chromatin assembly/disassembly. Further research will be necessary to determine if, for example, metabolic differences related to the *arginase 2* gene have been differentially selected in the lab and wild. Outlier analyses are susceptible to false positives because of bottlenecks and enhanced drift (Excoffier *et al.* 2009). Enhanced drift because of fluctuations in population size and breeding practices in lab strains cannot be rigorously ruled out as the cause of extreme differentiation without further investigation. Another leading candidate gene for the influence of domestication was *tpm3*, although it was associated with a synonymous substitution. This muscle contraction gene (Hsiao *et al.* 2003) may play a role in swimming demands faced by wild fish relative to domesticated strains. A muscle contraction gene plays a role in adaptive differentiation among whitefish species pairs (*Coregonus* spp.) that face different swimming demands (Derome *et al.* 2006).

### Genome-wide patterns of linkage disequilibrium

The striking difference in the overall pattern of LD in the AB lab strain compared with a wild population provides a clear example of the effects of domestication on locus interactions and has important implications for the influence of epistasis and adaptive processes in lab relative to wild populations. The high LD in the lab strain is likely caused by bottlenecks and inbreeding. Other domesticated and artificially selected populations have similarly high and wide-ranging LD, including dogs (Sutter *et al.* 2004; Gray *et al.* 2009), domestic sheep (McRae *et al.* 2002), pigs (Harmegnies *et al.* 2006), chickens (Heifetz *et al.* 2005), cattle (Khatkar *et al.* 2008) and thoroughbred horses (Tozaki *et al.* 2005). High levels of LD have also been observed in some wild populations such as an inbred Scandinavian wolf population (Bensch *et al.* 2006) and a population of bighorn sheep (Miller *et al.* 2011). In contrast, the extent of LD in natural zebrafish populations was markedly reduced compared with the lab strain and appeared to be lower than outbred wolf (Gray *et al.* 2009; vonHoldt *et al.* 2011) and flycatcher (Backstrom *et al.* 2006) populations, but more fine-scale analysis will be necessary to substantiate this comparison.

### The zebrafish as an ecological model

Our work establishes a foundation for studies that incorporate genetic variation from wild zebrafish populations to understand the full range of genetic influences on phenotypic variation in this species. Research on lab strains that incorporates genetic variation from wild populations will enhance understanding of modifiers of a rich array of existing mutants and genotype-phenotype associations discovered in lab strains. Research on the genetic architecture and adaptive significance of various traits in wild zebrafish populations will enhance understanding of genotype-phenotype associations under natural conditions. Further, this species occurs across a wide array of habitat types that vary in anthropogenic disturbance (e.g. pollution, fragmentation) and therefore offer opportunities for detailed analyses of adaptive evolution under a variety of ecological contexts. For example, zebrafish occur in a gradient of moving and still bodies of water (Spence *et al.* 2008) and could serve as a model for adaptive response to these conditions. The zebrafish is easily captured, can be maintained and bred easily in captivity or mesocosms, or can be studied under field conditions in Bangladesh, Nepal or India. Well-developed molecular genetic techniques such as morpholino gene knockdown could provide unprecedented analyses of a wide array of organismal effects of gene function in a vertebrate.

## Data accessibility

DNA sequences: Genbank accessions JN234180–JN234356.

SNP data: DRYAD entry doi:10.5061/dryad.505dp.

## Supporting information

**Fig. S1** Allele frequency spectra for wild populations and three lab strains.

**Fig. S2** Distribution of unbiased expectedheterozygosity (*H*_e_) values averaged over 10 wildpopulations (SRN and WYD excluded because of small sample size) orover three lab strains for SNPs.

**Fig. S3** Log probability from the STRUCTUREmodel against number of population groups (*K*).

**Table S1** Pairwise Φ_ST_ estimates(below diagonal), pairwise net nucleotide differences per site(*Da;* above diagonal, multiplied by100), and number of shared haplotypes between pairs of sample sites (in parentheses)

**Table S2** Pairwise genetic differentiation for SNPs

**Table S3** Outlier loci for 11 wild populations (lab strains excluded)

**Table S4** Outlier loci for 11 wild populations and three strains

**Data S1** Ascertainment bias.
