# Supplementary material for: Population genomics of wild and laboratory zebrafish (Danio rerio)
Source: Mol Ecol. 2011 Oct;20(20):4259–76. doi: 10.1111/j.1365-294X.2011.05272.x (PMC3627301; doi:10.1111/j.1365-294X.2011.05272.x)
Supplement: Supplementary file 1 [file mec0020-4259-SD1.pdf]

Supplemental Material for Ascertainment bias

We examined allele frequency spectra and the distribution of heterozygosity estimates to assess the degree to which ascertainment bias may influence our interpretations. Ascertainment bias did not appear to have a large influence on allele frequency spectra for the wild populations but did appear to have an influence on the lab strains (Fig. S1). For the wild populations, the lowest frequency minor allele class occurred at the highest frequency in all cases (Fig. S1). The allele frequency spectra for the AB and TM1 lab strains appeared biased towards moderate frequency alleles (Fig. S1). The SJA strain was developed as a highly inbred strain from the AB strain and this strain was generally depauperate of genetic variation, as expected. Heterozygosity within lab strains was also biased towards larger values (Fig. S2), but this was not the case in wild populations.

Our interpretations based on mtDNA sequence variation are free from ascertainment bias. However, it is possible that our inferences for the SNP data set were influenced by ascertainment bias. This set of SNPs was obtained using zebrafish from lab strains. Any bias associated with how these SNPs were developed could affect estimates of either a) within population (or lab strain) genetic diversity or b) genetic divergence among populations (or lab strains). We expect an upward bias for estimates of within-lab strain genetic diversity. We do not *a priori* expect bias in estimates of within-population genetic variation of wild populations, because the SNPs were developed independently of the wild fish. Indeed, the lowest frequency minor allele class did appear to be underrepresented and heterozygosity appeared to be biased towards larger values in the lab strains examined (Figs. S1 & S2). However, within-population genetic variation of

wild populations did not appear to be strongly influenced by ascertainment bias based on the distribution of minor allele frequencies and heterozygosity (Figs. S1 & S2).

Estimates of among-population (or lab strain) genetic divergence and analysis of outlier loci could also be influenced by ascertainment bias. Use of a geographically restricted ascertainment panel causes an analysis to miss polymorphisms that exist exclusively outside the panel populations (Smith *et al.* 2007). In our study, we were unable to examine polymorphisms that exist exclusively in wild populations and therefore, estimate of genetic differentiation may be biased low. Furthermore, low frequency alleles that are the result of recent mutation are expected to cluster around the geographic location where the mutation first arose (Novembre *et al.* 2008). This type of variation would also go undetected in our analysis, further contributing to downward bias in estimates of genetic differentiation and limiting our ability to detect regional genetic substructure. Furthermore, rare alleles potentially involved in selection are likely to be underrepresented in our SNP panel, thus reducing power to detect natural selection (Namroud *et al.* 2008). These factors reduce the likelihood that we would detect genetic structure and evidence of natural selection in our dataset. Further analysis of zebrafish wild populations with resequencing techniques may uncover further subdivision and further evidence of the influence of natural selection than we observed in the present study. However, our interpretations based on the relative level of among-population genetic divergence for the SNP panel we used should not be prone to false positive detection of genetic differentiation, especially since any bias is expected to be towards substructure or signatures of selection going undetected.

Literature Cited

Namroud MC, Beaulieu J, Juge N, Laroche J, Bousquet J (2008) Scanning the genome for gene single nucleotide polymorphisms involved in adaptive population differentiation in white spruce. *Molecular Ecology* **17**, 3599-3613.

Novembre J, Johnson T, Bryc K*, et al.* (2008) Genes mirror geography within Europe. *Nature* **456**, 98-U95.

Smith CT, Antonovich A, Templin WD*, et al.* (2007) Impacts of marker class bias relative to locus-specific variability on population inferences in Chinook salmon: A comparison of single-nucleotide polymorphisms with short tandem repeats and allozymes. *Transactions of the American Fisheries Society* **136**, 1674-1687.

Table S1. Pairwise Φ_{ST} estimates (below diagonal), pairwise net nucleotide differences per site (*Da;* above diagonal, multiplied by 100), and number of shared haplotypes between pairs of sample sites (in parentheses). Values of Φ_{ST} in bold were significant based on 10,000 permutations and following FDR correction ($\alpha = 0.05$).

ID	PAR	KHA	BER	SHK	JOR	PGM	PNS	UTR	RCH	CHT	SRN	WYD	AB	SJA	TM1
PAR	--	5.48 (0)	0.31 (0)	0.18 (0)	0.28 (0)	0.23 (0)	0.22 (0)	0.21 (0)	4.13 (0)	4.47 (0)	6.71 (0)	5.93 (0)	0.48 (0)	0.48 (0)	0.39 (0)
KHA	**0.908**	--	5.61 (0)	5.62 (0)	5.70 (0)	5.64 (0)	5.56 (0)	5.47 (0)	5.34 (0)	5.46 (0)	7.58 (0)	6.73 (0)	5.57 (0)	5.57 (0)	5.67 (0)
BER	**0.327**	**0.947**	--	0.033 (1)	0.04 (1)	0.02 (1)	0.02 (1)	0.08 (1)	4.12 (0)	4.44 (0)	6.82 (0)	5.97 (0)	0.10 (0)	0.10 (0)	0.01 (1)
SHK	**0.192**	**0.941**	0.073	--	0.017 (3)	-0.03 (3)	-0.01 (2)	0.01 (1)	4.04 (0)	4.33 (0)	6.66 (0)	5.92 (0)	0.19 (0)	0.19 (0)	0.10 (1)
JOR	**0.328**	**0.958**	**0.104**	0.056	--	0.01 (3)	0.04 (1)	0.08 (1)	4.07 (0)	4.38 (0)	6.75 (0)	5.96 (0)	0.16 (0)	0.16 (0)	0.07 (1)
PGM	**0.239**	**0.937**	0.049	-0.052	0.032	--	-0.01 (1)	0.02 (1)	4.03 (0)	4.33 (0)	6.64 (0)	5.98 (0)	0.17 (0)	0.17 (0)	0.08 (1)
PNS	**0.235**	**0.934**	0.031	-0.027	0.088	-0.009	--	0.01 (2)	4.06 (0)	4.38 (0)	6.72 (0)	5.95 (0)	0.16 (0)	0.16 (0)	0.07 (1)
UTR	**0.219**	**0.927**	**0.141**	0.020	**0.158**	0.042	0.015	--	3.95 (0)	4.30 (0)	6.59 (0)	5.88 (0)	0.24 (1)	0.24 (1)	0.16 (1)
RCH	**0.792**	**0.877**	**0.832**	**0.804**	**0.843**	**0.811**	**0.816**	**0.807**	--	0.64 (0)	3.46 (0)	3.21 (0)	4.36 (0)	4.36 (0)	4.26 (0)
CHT	**0.867**	**0.937**	**0.909**	**0.895**	**0.921**	**0.893**	**0.893**	**0.885**	**0.421**	--	3.38 (0)	3.11 (0)	4.67 (0)	4.67 (0)	4.58 (0)
SRN	**0.895**	**0.973**	**0.951**	**0.939**	**0.967**	**0.933**	**0.932**	**0.923**	**0.744**	**0.885**	--	4.19 (0)	7.06 (0)	7.06 (0)	6.95 (0)
WYD	**0.874**	**0.967**	**0.940**	**0.923**	**0.960**	**0.919**	**0.918**	**0.908**	**0.707**	**0.866**	0.993	--	6.22 (0)	6.22 (0)	6.12 (0)
AB	**0.464**	**0.975**	**0.279**	**0.414**	**0.504**	**0.340**	**0.301**	**0.373**	**0.850**	**0.940**	**1.000**	**0.999**	--	0 (1)	0.09 (0)
SJA	**0.464**	**0.975**	**0.279**	**0.414**	**0.504**	**0.340**	**0.301**	**0.373**	**0.850**	**0.940**	**1.000**	**0.999**	0	--	0.09 (0)
TM1	**0.410**	**0.975**	0.016	**0.267**	**0.307**	**0.186**	**0.141**	**0.280**	**0.847**	**0.938**	**1.000**	**0.999**	**1.000**	**1.000**	--

Table S2. Pairwise genetic differentiation for SNPs. D_{est} is above diagonal and F_{ST} is below the diagonal. The only non-significant results are shown in bold (P = 0.59 for D_{est}, P = 0.72 for F_{ST}). All other values were significant after controlling the FDR (α = 0.05).

ID	PAR	KHA	BER	DHO	JOR	PGM	PNS	UTR	RCH	CHT	SRN	WYD	AB	SJA	TM1
PAR	--	0.123	0.098	0.099	0.094	0.094	0.085	0.087	0.098	0.145	0.156	0.160	0.146	0.160	0.142
KHA	0.475	--	0.114	0.110	0.112	0.108	0.104	0.114	0.111	0.119	0.103	0.108	0.164	0.178	0.176
BER	0.270	0.379	--	0.007	0.007	0.008	0.028	0.026	0.022	0.100	0.128	0.125	0.107	0.119	0.097
DHO	0.283	0.397	0.021	--	0.005	0.005	0.027	0.024	0.019	0.099	0.127	0.127	0.098	0.121	0.090
JOR	0.260	0.380	0.018	0.015	--	**-0.001**	0.025	0.025	0.011	0.098	0.123	0.121	0.100	0.122	0.087
PGM	0.259	0.367	0.021	0.018	**-0.002**	--	0.026	0.025	0.014	0.095	0.125	0.120	0.099	0.124	0.090
PNS	0.221	0.324	0.069	0.072	0.061	0.064	--	0.012	0.031	0.102	0.120	0.118	0.097	0.127	0.086
UTR	0.227	0.351	0.066	0.064	0.061	0.062	0.027	--	0.032	0.103	0.127	0.122	0.098	0.129	0.082
RCH	0.282	0.405	0.059	0.056	0.032	0.040	0.077	0.080	--	0.084	0.126	0.121	0.105	0.130	0.099
CHT	0.505	0.610	0.338	0.355	0.337	0.326	0.309	0.315	0.319	--	0.128	0.112	0.159	0.167	0.172
SRN	0.477	0.639	0.315	0.334	0.308	0.311	0.268	0.278	0.332	0.640	--	0.072	0.180	0.188	0.197
WYD	0.461	0.631	0.289	0.310	0.280	0.281	0.245	0.246	0.298	0.586	0.856	--	0.193	0.192	0.190
AB	0.434	0.588	0.292	0.285	0.281	0.277	0.250	0.254	0.302	0.559	0.555	0.551	--	0.091	0.125
SJA	0.571	0.783	0.404	0.436	0.417	0.416	0.381	0.392	0.465	0.746	0.880	0.872	0.455	--	0.173
TM1	0.391	0.604	0.237	0.232	0.214	0.223	0.194	0.181	0.250	0.572	0.511	0.453	0.370	0.654	--

Table S3. Outlier loci for 11 wild populations (lab strains excluded). Bold rows are those that were signficant outliers in the analysis with and without lab strains. Chromosome and map positions correspond to build Zv9, the 9th build of the zebrafish genome. Asterisks represent significant reduction in heterozygosity or elevation in LD within 10 Mb windows surrounding outliers ($P < 0.05$). We used the AmiGO browser of gene ontology (www.geneontology.org), the KEGG PATHWAY database (www.genome.jp/kegg/pathway.html), and the UniProt database (www.expasy.uniprot.org), along with corresponding literature searches to assign significant outlier SNPs to putative functional groups. Rows with red text represent significant clusters of loci.

SS Number	RS Number	Chromosome	Map Position	Heterozygosity 10 Mb Window	LD in 10 Mb Window	Observed Fst	Fst *P*-value	Substitution Type	Gene Abbreviation	Gene	AmiGO Gene Ontology
High Fst outliers											
ss49835357	rs41024117	1	22987424	0.147	--	0.815	0.004	synonymous	im:7138144	Unknown	
ss49818106	rs41049628	1	26830002	0.089*	0.007	0.730	0.003	synonymous	pspc1	paraspeckle component 1	regulation of transcription
**ss49816566**	**rs40688913**	**1**	**51201608**	0.156	0.007	**0.841**	**0.000**	**synonymous**	**Unknown**		
ss49812317	rs40993451	1	54591731	0.169	--	0.728	0.003	synonymous	dla	deltaA	Notch signalling pathway, cell signalling
**ss49835326**	**rs41033476**	**1**	**60346317**	0.113	--	**0.965**	**0.000**	**Unknown**	**Unknown**		
ss49818986	rs40632210	2	3895625	0.136	--	0.673	0.008	nonsynonymous	zgc:153222	Unknown	
ss49818617	rs41053775	2	7511351	0.102	0.014	0.708	0.004	synonymous	eif4a2	eukaryotic translation initiation factor 4A, isoform 2	ATP binding
**ss49791139**	**rs41126962**	**2**	**9315232**	0.088*	0.007	**0.721**	**0.004**	**nonsynonymous**	wu:fi34b01	Unknown	
**ss49799181**	**rs40689405**	**3**	**35617930**	0.095	--	**0.939**	**0.000**	**synonymous**	**Unknown**		
ss49795042	rs40724784	5	2513344	0.154	0.010	0.654	0.008	synonymous	hspa5	heat shock protein 5	response to stress
ss49838125	rs41209191	5	65387878	0.123	0.021	0.683	0.009	synonymous	pho	phoenix	neuromast regeneration
ss49817325	rs41226086	5	70994674	0.130	0.014	0.737	0.004	nonsynonymous	Unknown		
ss49816963	rs41245927	5	73132173	0.161	0.005	0.709	0.003	synonymous	Unknown		
ss49819511	rs40951048	6	13300691	0.170	0.020	0.886	0.001	synonymous	Unknown		
ss49817812	rs41139565	7	30387884	0.151	0.015	0.777	0.000	nonsynonymous	gfod2	glucose-fructose oxidoreductase domain containing 2	metabolic process
ss49808440	rs40648515	7	34680961	0.171	0.011	0.691	0.004	synonymous	anp32a	acidic (leucine-rich) nuclear phosphoprotein 32 family, member A	protein binding
ss49815160	rs41246373	8	50701507	0.168	--	0.682	0.007	synonymous	prdm16	PR domain containing 16	nucleic acid binding
ss49818125	rs40966567	9	3913173	0.275	--	0.789	0.002	synonymous	ubr3	ubiquitin protein ligase E3 component n-recognin 3	metal ion binding
ss49808556	rs41188722	9	25256497	0.156	0.010	0.707	0.007	synonymous	Q7SZN3_DANRE	Unknown	
ss49824496	rs41019193	9	28725883	0.146	0.020	0.685	0.006	synonymous	***si:ch211-194d6.2***	**regulation of transcription**	
ss49828807	rs40886898	9	57959194	0.210	0.014	0.665	0.005	synonymous	zgc:163002	Unknown	
**ss49813621**	**rs40824798**	**10**	**1739884**	0.155	0.015	**0.718**	**0.003**	**synonymous**	**Unknown**		
ss49797454	rs41227753	10	16063703	0.181	--	0.671	0.008	synonymous	phax	Unknown	
ss49839056	rs40994501	10	24871019	0.183	0.013	0.738	0.003	synonymous	zgc:158234	regulation of transcription	
ss49819959	rs40636226	11	6361679	0.208	0.011	0.681	0.004	synonymous	Unknown		
ss49811453	rs40808417	14	9600057	0.139	0.021	0.734	0.006	synonymous	NP_001154808.1	Unknown	
ss49823154	rs41192588	14	14553656	0.154	0.015	0.701	0.005	synonymous	Unknown		
ss49833164	rs40929442	14	25332481	0.179	0.009	0.691	0.006	nonsynonymous	zgc:158426		signal transduction
ss49821286	rs40738574	14	26176485	0.171	0.027	0.767	0.002	synonymous	Unknown		
ss49813166	rs41215491	14	27459642	0.186	0.014	0.679	0.009	synonymous	gpr137	G protein-coupled receptor 137	receptor activity
ss49831087	rs41249938	14	30652433	0.183	0.028	0.739	0.004	synonymous	Unknown		
ss49835823	rs40736428	16	45145746	0.153	0.008	0.771	0.003	synonymous	Unknown		
ss49836611	rs40647130	16	57279583	0.132	0.017	0.773	0.001	synonymous	Unknown		
**ss49805461**	**rs40665250**	**17**	**734651**	0.083*	0.005	**0.750**	**0.002**	**synonymous**	**Unknown**		
ss49832807	rs41040746	17	15378638	0.120	--	0.834	0.002	synonymous	marcksb	myristoylated alanine rich protein kinase C substrate b	calmodulin binding, kinase activity, calcium binding
ss49838165	rs41140735	17	51782410	0.154	0.015	0.800	0.001	synonymous	ttc15	tetratricopeptide repeat domain 15	cellular component unknown
ss49806727	rs41217039	18	345072	0.272	0.021	0.699	0.004	synonymous	larp6	La ribonucleoprotein domain family, member 6	RNA binding
**ss49822232**	**rs40610581**	**18**	**39879510**	0.197	0.032	**0.898**	**0.001**	**synonymous**	**selt1b**	**Selenoprotein T1b Precursor**	**regulation of cell redox homeostasis**
**ss49813231**	**rs40782513**	**18**	**45753702**	0.129	--	**0.643**	**0.010**	**synonymous**	**zgc:77304**	**RNA binding motif protein 7**	**nucleic acid binding**
ss49811414	rs41127410	19	8100695	0.180	--	0.741	0.003	synonymous	fam63a	family with sequence similarity 63, member A	
**ss49820695**	**rs40770657**	**19**	**11924194**	0.161	--	**0.747**	**0.003**	**synonymous**	**Unknown**		
**ss49826750**	**rs41099232**	**19**	**16724865**	0.178	--	**0.728**	**0.002**	**synonymous**	**sf3a3**	**splicing factor 3a, subunit 3**	**nucleic acid bindng**
ss49825575	rs40846537	19	20365636	0.200	0.009	0.691	0.005	synonymous	Unknown		
ss49814086	rs40916406	19	34341885	0.137	--	0.796	0.001	synonymous	atp6v1c1b	ATPase, H+ transporting, lysosomal, V1 subunit C, isoform 1b	energetic metabolism
ss49810528	rs41197719	20	838989	0.188	--	0.684	0.007	synonymous	si:ch211-241p10.1	Unknown	
**ss49799497**	**rs40610352**	**20**	**19508549**	0.127	--	**0.837**	**0.000**	**synonymous**	**Unknown**		
ss49816977	rs40802613	20	38549596	0.133	0.015	0.718	0.004	synonymous	si:ch211-245h14.1	Unknown	
**ss49833927**	**rs40604164**	**20**	**43415633**	0.149	--	**0.672**	**0.006**	**nonsynonymous**	**si:dkey-14a7.1**	**protein unc-93 homolog A**	**Unknown**
ss49820804	rs40700276	20	53192595	0.245	0.026	0.657	0.008	synonymous	Unknown		
ss49837046	rs40625276	21	7526282	0.202	--	0.713	0.008	synonymous	otpa	orthopedia homolog a	DNA binding
**ss49816753**	**rs41046240**	**21**	**9670969**	0.213	--	**0.723**	**0.009**	**synonymous**	**Unknown**		
**ss49798351**	**rs41210614**	**22**	**592861**	0.178	--	**0.758**	**0.003**	**synonymous**	**sfrs3b**	**serine/arginine-rich splicing factor 3b**	**nucleic acid bindng**
**ss49816041**	**rs41136060**	**23**	**26760166**	0.189	0.016	**0.833**	**0.001**	**synonymous**	**Unknown**		
ss49808936	rs40801290	23	41579833	0.088*	0.016	0.642	0.008	Unknown	Unknown		
ss49807899	rs41219652	23	44979575	0.080*	--	0.665	0.009	synonymous	Unknown		
ss49834203	rs41112794	25	32474214	0.114	0.005	0.725	0.009	nonsynonymous	Unknown		
ss49832161	rs40938340	--	--	--	--	0.760	0.002	Unknown	Unknown		
ss49837615	rs41217016	--	--	--	--	0.716	0.003	Unknown	Unknown		
ZSNP642	rs3728107	--	--	--	--	0.670	0.005	Unknown	Unknown		
**ss49837791**	**rs40623529**	**--**	**--**	**--**	--	**0.785**	**0.001**	**Unknown**	**Unknown**		
ss49823178	rs41151973	--	--	--	--	0.827	0.002	Unknown	Unknown		
ss49839306	--	--	--	--	--	0.762	0.001	Unknown	Unknown		

Low Fst outliers											
**ss49816649**	**rs41204411**	**2**	**40583543**	0.184	0.016	**0.026**	**0.009**	**synonymous**	**Unknown**		
ss49802804	rs40944649	5	40755171	0.213	0.038*	-0.005	0.001	synonymous	paqr3a	progestin and adipoQ receptor family member IIIa	receptor activity
**ss49838285**	**rs40690148**	**8**	**37759416**	0.119	0.018	**-0.003**	**0.001**	**Unknown**	**Unknown**		
ss49830662	rs40973120	11	34344146	0.167	0.027	0.009	0.002	synonymous	LOC798019	Unknown	
ss49811048	rs40983990	20	3602475	0.139	0.005	0.023	0.010	synonymous	Unknown		
ss49789210	rs40867301	20	34365708	0.112	0.013	0.019	0.009	nonsynonymous	ivns1abpa	influenza virus NS1A binding protein a	immune function
ss49816863	rs40668620	21	21961892	0.076*	0.016	0.025	0.005	synonymous	birc2	baculoviral IAP repeat-containing 2	regulation of apoptosis
**ZSNP1493**	**--**	**--**	**--**			**0.022**	**0.005**	**Unknown**	**--**		
**ss49821086**	**rs40887001**	**--**	**--**			**0.022**	**0.004**	**Unknown**	**Unknown**		

Table S4. Outlier loci for 11 wild populations and three strains. Bold rows are those that were signficant outliers in the analysis with and without lab strains. Map positions correspond to Zv9, the 9th build of the zebrafish genome. Asterisks represent significant reduction in heterozygosity or elevation in LD within 10 Mb windows surrounding outliers ($P < 0.05$). We used the AmiGO browser of gene ontology (www.geneontology.org), the KEGG PATHWAY database (www.genome.jp/kegg/pathway.html), and the UniProt database (www.expasy.uniprot.org), along with corresponding literature searches to assign significant outlier SNPs to putative functional groups. Rows with red text represent significant clusters of loci.

SS Number	RS Number	Chromosome	Map Position	Expected Heterozygosity 10 Mb Window - AB	LD in 10 Mb Window	Observed Fst	Fst *P*-value	Substitution Type	Gene Abbreviation	Gene	AmiGO Gene Ontology
High Fst outliers											
ss49801948	rs3727476	1	494991	0.134	--	0.902	0.000	synonymous	Unknown		
ss49815455	rs40870143	1	30597323	0.000*	--	0.878	0.002	Unknown	Unknown		
ss49792989	rs40939599	1	44430930	0.000*	--	0.985	0.000	Unknown	Unknown		
**ss49816566**	**rs40688913**	**1**	**51201608**	0.069	--	**0.867**	**0.002**	**synonymous**	**Unknown**		
**ss49835326**	**rs41033476**	**1**	**60346317**	0.224	0.512	**0.901**	**0.000**	**Unknown**	**Unknown**		
**ss49791139**	**rs41126962**	**2**	**9315232**	0.061	--	**0.828**	**0.002**	**nonsynonymous**	**Unknown**		
ss49817314	rs41122823	3	26601927	0.184	--	0.870	0.003	synonymous	Unknown		
**ss49799181**	**rs40689405**	**3**	**35617930**	0.078	--	**0.935**	**0.004**	**synonymous**	**Unknown**		
ss49814002	rs40597362	3	42780210	0.100	--	0.878	0.002	synonymous	litaf	Lipopolysaccharide-induced tumor necrosis factor-alpha factor homolog	regulation of transcription
ss49829527	rs40932613	4	7225892	0.151	--	0.950	0.000	synonymous	Unknown		
ss49806742	rs40962913	4	9877893	0.142	--	0.897	0.003	synonymous	Unknown		
ss49814527	rs40886238	5	43422339	0.104	--	0.878	0.001	nonsynonymous	si:dkey-65b12.2-001		Oxidoreductase activity
ss49787521	rs41000902	6	34893055	0.122	--	0.856	0.005	synonymous	serbp1	SERPINE1 mRNA binding protein 1	nucleic acid binding
ss49789521	rs40845657	6	34893249	0.122	--	0.825	0.009	synonymous	serbp1	SERPINE1 mRNA binding protein 1	nucleic acid binding
ss49830266	rs41087033	6	39684725	0.130	0.431	0.784	0.009	synonymous	pfkmb	phosphofructokinase, muscle b	metabolic processes
ss49812055	rs40626431	7	18940707	0.047	0.094	0.957	0.001	synonymous	men1	multiple endocrine neoplasia type 1	regulation of transcription
ZSNP564	rs3728037	7	24083838	0.082	--	0.814	0.007	synonymous	tnfsf10l	tumor necrosis factor (ligand) superfamily, member 10 like	immune response; induction of apoptosis
ss49813207	rs40609782	7	25628206	0.094	--	0.841	0.006	synonymous	Unknown		
ss49825036	rs41208967	7	39787518	0.162	0.205	0.884	0.001	nonsynonymous	ccdc123	Coiled-coil domain-containing protein 123, mitochondrial Precursor	Unknown
ss49814608	rs40936835	7	40568892	0.166	0.209	0.896	0.001	synonymous	bhlhe40	basic helix-loop-helix family, member e40	regulation of transcription; circadian rhythms in mammals
ss49810750	rs40866434	7	53973893	0.026*	--	0.921	0.001	synonymous	tcf12	transcription factor 12	regulation of transcription
ss49812607	rs41040388	9	28427920	0.091	--	0.972	0.001	nonsynonymous	si:dkey-22a1.2		Unknown
ss49813456	rs40627245	9	29378025	0.079	0.707*	0.830	0.005	synonymous	klf7l	Kruppel-like factor 7	nucleic acid binding
ss49807961	rs41231373	9	30750258	0.077	0.836*	0.956	0.002	synonymous	Unknown		
**ss49813621**	**rs40824798**	**10**	**1739884**	0.093	--	**0.773**	**0.010**	**synonymous**	**Unknown**		
ss49839551	rs41095323	11	8769420	0.239	--	0.802	0.006	synonymous	Unknown		
ss49806479	rs41081072	11	28260424	0.221	0.517	0.836	0.006	synonymous	fbln2	Fibulin 2 Fragment	calcium ion binding
ss49821058	rs40675988	11	36600920	0.196	--	0.896	0.001	synonymous	Unknown		
ss49815402	rs40671280	11	36883683	0.203	--	0.880	0.001	synonymous	zgc:86903	Probable protein BRICK1	regulation of actin cytoskeleton
ss49795626	rs40943923	11	36889436	0.203	--	0.806	0.008	synonymous	gpx1a	glutathione peroxidase 1	metabolic process; glutathione metabolism
ss49800774	rs40977791	12	9333803	0.080	--	0.952	0.002	synonymous	nrbf2	nuclear receptor binding factor 2	signal transducer activity; receptor activity
ss49803445	rs41191772	13	11762027	0.078	0.424	0.906	0.001	synonymous	Unknown		
ss49793380	rs40828248	13	24608440	0.000*	--	0.832	0.007	synonymous	tbp	TATA-box-binding protein (zTBP)	regulation of transcription
ss49810074	rs40874994	13	28725137	0.070	--	0.812	0.010	synonymous	fgf8b	fibroblast growth factor 8 a	growth factor activity
ss49798502	rs40870913	13	28899556	0.073	--	0.947	0.001	nonsynonymous	inaa	internexin neuronal intermediate filament protein, alpha	intermediate filament associated protein
ss49808791	rs40802740	13	33257060	0.166	0.255	0.768	0.009	nonsynonymous	arg2	arginase 2	metabolic processes
ss49807345	rs41024937	14	1210170	0.132	--	0.847	0.004	synonymous	zgc:153259	ADP-ribosylation factor-like 9	nucleic acid binding; GTP binding
ss49823599	rs40673921	14	33486637	0.247	0.130	0.886	0.003	synonymous	Unknown		
ss49823250	rs41129224	14	45217625	0.107	0.292	0.892	0.003	synonymous	pcdh10b	protocadherin 10b	cell adhesion
ss49808026	rs40829752	15	18856441	0.075	0.166	0.786	0.010	synonymous	Unknown		
ss49816067	rs40857229	15	46227314	0.166	--	0.933	0.001	Unknown	Unknown		
ss49817272	rs40592636	16	9600741	0.275	--	0.807	0.009	synonymous	Unknown		
ss49790328	rs40848143	16	54740013	0.205	--	0.904	0.001	synonymous	zgc:123210	high-mobility group nucleosomal binding domain 2-like	nucleic acid binding
**ss49805461**	**rs40665250**	**17**	**734651**	0.121	--	**0.745**	**0.008**	**synonymous**	**Unknown**		
ss49807844	rs40992995	17	11256397	0.252	0.256	0.871	0.002	nonsynonymous	arid4a	arid4a AT rich interactive domain 4A (RBP1-like)	chromatin assembly/disassembly
ss49832361	rs40654714	18	39453913	0.126	0.220	0.941	0.002	synonymous	dmxl2	Dmx-like 2	GTPase bindng; Rab escort protein activity
**ss49822232**	**rs40610581**	**18**	**39879510**	0.133	0.162	**0.846**	**0.004**	**synonymous**	**selt1b**	**Selenoprotein T1b Precursor**	**cell redox homeostasis; selenium binding**
**ss49813231**	**rs40782513**	**18**	**45753702**	0.034	--	**0.772**	**0.006**	**synonymous**	**zgc:77304**	**RNA binding motif protein 7**	**nucleic acid binding**
ss49819780	rs40935300	19	5863954	0.140	--	0.790	0.010	synonymous	syntaxin1b	syntaxin1b	cell signalling; intracellular protein transport
ss49819910	rs40710311	19	9274627	0.117	--	0.819	0.007	synonymous	Unknown		
ss49792329	rs41008636	19	11478953	0.045	--	0.890	0.001	synonymous	tpm3	tropomyosin 3 isoform 2	muscle contraction; actin binding
**ss49820695**	**rs40770657**	**19**	**11924194**	0.036	--	**0.852**	**0.001**	**synonymous**	**Unknown**		
**ss49826750**	**rs41099232**	**19**	**16724865**	0.020	--	**0.811**	**0.004**	**synonymous**	**sf3a3**	**splicing factor 3a, subunit 3**	**nucleic acid bindng**
ss49839514	rs40818443	20	4754699	0.133	--	0.964	0.000	synonymous	papolb	poly(A) polymerase beta (testis specific)	nucleic acid binding
ss49826855	rs41142993	20	7526149	0.165	0.795*	0.826	0.006	synonymous	Unknown		
**ss49799497**	**rs40610352**	**20**	**19508549**	0.106	--	**0.829**	**0.006**	**synonymous**	**Unknown**		
ss49815119	rs40917760	20	23842885	0.071	0.772*	0.818	0.005	synonymous	si:dkey-15j16.2	Uncharacterized protein C6orf72 homolog Precursor	cell signalling
**ss49833927**	**rs40604164**	**20**	**43415633**	0.114	--	**0.762**	**0.010**	**nonsynonymous**	**si:dkey-14a7.1**	**protein unc-93 homolog A**	**Unknown**
**ss49816753**	**rs41046240**	**21**	**9670969**	0.100	--	**0.852**	**0.002**	**synonymous**	**Unknown**		

ss49811482	rs40909712	21	11964629	0.092	--	0.903	0.000	synonymous	Unknown		
ss49818433	rs40838644	21	40923753	0.218	--	0.810	0.007	synonymous	Unknown		
**ss49798351**	**rs41210614**	**22**	**592861**	0.190	--	**0.814**	**0.003**	**synonymous**	**sfrs3b**	**splicing factor, arginine/serine-rich 3b**	**nucleic acid binding**
ss49821842	rs40597197	22	20611769	0.239	--	0.825	0.009	synonymous	mbd3b	methyl-CpG binding domain protein 3b	nucleic acid binding
ZSNP1744	rs3729206	22	32554333	0.100	--	0.845	0.006	synonymous	smc3	chondroitin sulfate proteoglycan 6	ATP binding
ss49832282	rs40654219	23	24588373	0.159	--	0.784	0.002	synonymous	Unknown		
**ss49816041**	**rs41136060**	**23**	**26760166**	0.098	0.742*	**0.818**	**0.008**	**synonymous**	**Unknown**		
ss49832469	rs40794973	23	36186124	0.054	--	0.901	0.000	synonymous	hoxc3a	homeo box C3a	regulation of transcription
ss49814397	rs40840492	25	2227095	0.152	--	0.813	0.009	synonymous	Unknown		
ss49821707	rs40737345	25	5666097	0.139	--	0.726	0.009	synonymous	Unknown		
ss49806323	rs41012997	25	37346409	0.102	0.216	0.796	0.009	synonymous	Unknown		
ZSNP652	rs3728117	--	--	--	--	0.916	0.000	Unknown	Unknown		
**ss49837791**	**rs40623529**	**--**	**--**	**--**	--	**0.865**	**0.000**	**Unknown**	**Unknown**		
ss49834489	rs40966784	--	--	--	--	0.755	0.009	Unknown	Unknown		
ss49837833	rs40609297	--	--	--	--	0.890	0.001	Unknown	Unknown		
ss49834474	rs41038387	--	--	--	--	0.799	0.008	Unknown	Unknown		
Low Fst outliers											
**ss49816649**	**rs41204411**	**2**	**40583543**	0.107	--	**0.052**	**0.002**	**synonymous**	**Unknown**		
ss49810958	rs40629453	6	7574608	0.070	0.045	0.057	0.002	synonymous	rpsa	ribosomal protein SA	receptor activity
ss49807929	rs40798839	6	13779754	0.162	--	0.072	0.004	synonymous	zgc:112212	Transmembrane protein 198	Unknown
ss49820991	rs40708264	6	37424300	0.119	0.335	0.086	0.005	synonymous	Unknown		
ss49817221	rs40917990	7	19833010	0.064	0.075	0.101	0.008	synonymous	coro1b	coronin, actin binding protein, 1B	actin binding
**ss49838285**	**rs40690148**	**8**	**37759416**	0.064	0.538	**0.020**	**0.000**	**Unknown**	**Unknown**		
ss49808817	rs40947847	8	50252170	0.168	--	0.077	0.003	synonymous	Unknown		
ss49792854	rs40788149	9	42160986	0.222	0.228	0.095	0.005	nonsynonymous	asnsd1	Asparagine synthetase domain-containing protein 1	metabolic processes; asparagine biosynthetic process
ss49837113	rs40906750	10	14457969	0.152	0.606*	0.085	0.004	synonymous	sigmar1	Sigma 1-type opioid receptor	transport; lipid transport
ss49813495	rs41230365	10	20181385	0.231	--	0.107	0.010	synonymous	epb4.9	erythrocyte membrane protein band 4.9 (dematin)	Unknown
ss49813006	rs40752287	12	18705985	0.278	--	0.079	0.008	synonymous	dlgap5	discs, large *(Drosophila)* homolog-associated protein 5	cell signalling
ss49799057	rs41104599	13	31041921	0.118	0.595*	0.075	0.002	synonymous	Unknown		
ss49821325	rs40743853	13	39492467	0.223	0.067	0.094	0.008	Unknown	Unknown		
ss49828424	rs40904718	13	53978736	0.067	--	0.062	0.003	synonymous	Unknown		
ss49821061	rs41103549	18	37334865	0.156	0.036	0.089	0.005	synonymous	arpp19a	cAMP-regulated phosphoprotein 19a	metabolic processes; regulation of glucose metabolism
**ZSNP1493**	**rs3728957**	**19**	**7628145**	0.119	--	**0.057**	**0.001**	**Unknown**	**--**		
ss49825781	rs41142032	19	41097376	0.140	0.035	0.082	0.007	synonymous	si:ch211-173p18.2	Novel protein Fragment	Unknown
ss49816435	rs40826660	20	25300346	0.071	**0.772***	0.060	0.003	synonymous	moxd1	DBH-like monooxygenase protein 1 homolog Precursor	metabolic processes
ss49826757	rs41016422	21	11634466	0.096	0.188	0.039	0.001	synonymous	Unknown		
ss49812670	rs40901098	22	836792	0.190	0.081	0.089	0.008	synonymous	zgc:103487	DENN/MADD domain containing 2D	Unknown
ss49824214	rs40685695	22	8787600	0.197	0.281	0.064	0.001	synonymous	si:dkey-182g1.2	hypothetical protein LOC100005482	Unknown
ss49821490	rs40878237	22	30351742	0.112	--	0.095	0.009	synonymous	si:ch211-239j15.4	hypothetical protein LOC100006377	Unknown
ss49822596	rs40768893	23	3531285	0.159	0.263	0.059	0.002	synonymous	spata2	spermatogenesis associated 2	reproductive process; spermatogenesis
**ss49821086**	**rs40887001**	**--**	**--**	**--**	**--**	**0.029**	**0.000**	**Unknown**	**Unknown**		

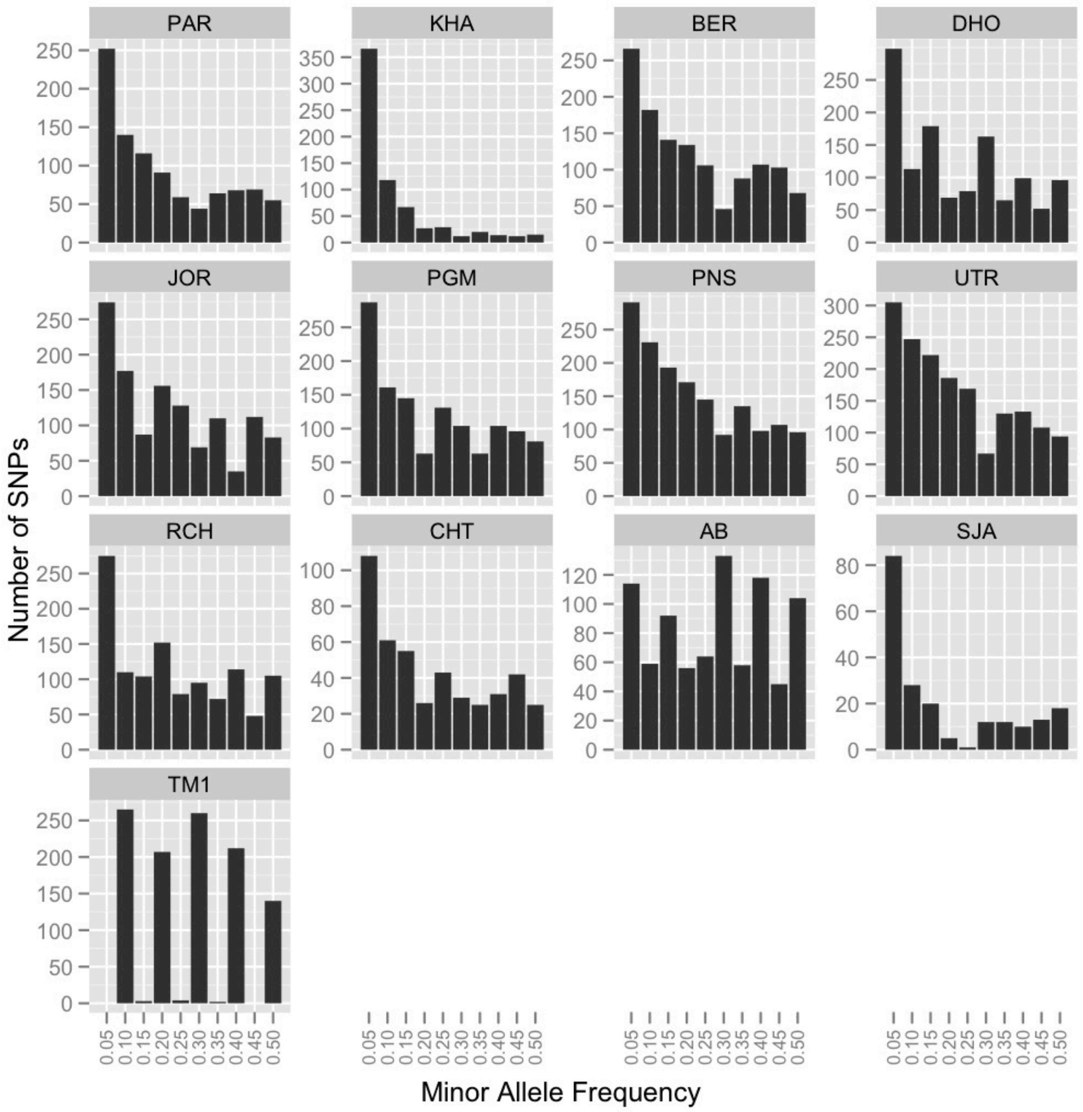
PAR
KHA
BER
DHO
JOR
PGM
PNS
UTR
RCH
CHT
AB
SJA
TM1
Number of SNPs
Minor Allele Frequency
0.05
0.10
0.15
0.20
0.25
0.30
0.35
0.40
0.45
0.50

Fig. S1

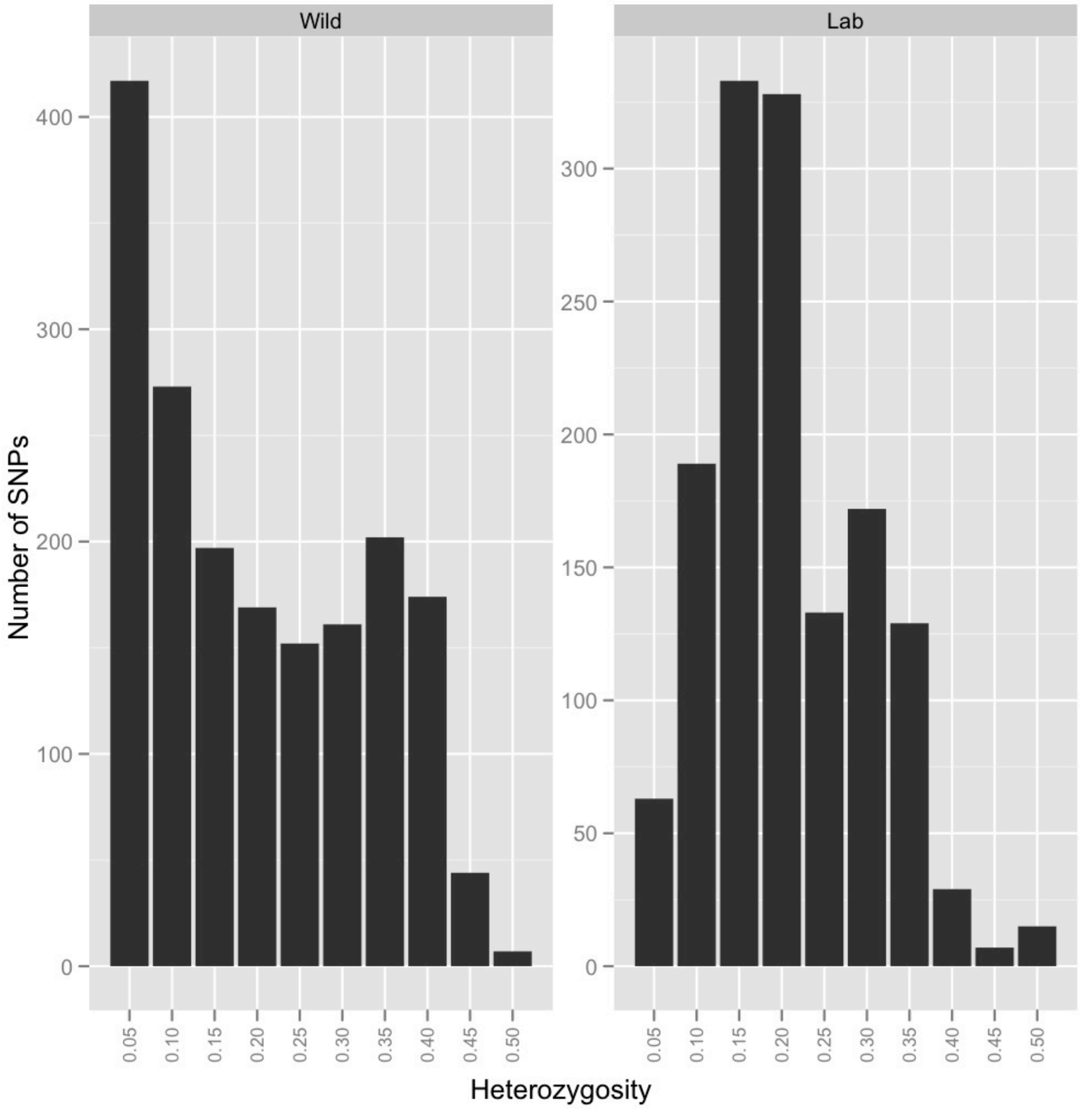

Wild
Lab
Number of SNPs
0
100
200
300
400
0
50
100
150
200
250
300
0.05
0.10
0.15
0.20
0.25
0.30
0.35
0.40
0.45
0.50
Heterozygosity

Fig. S2

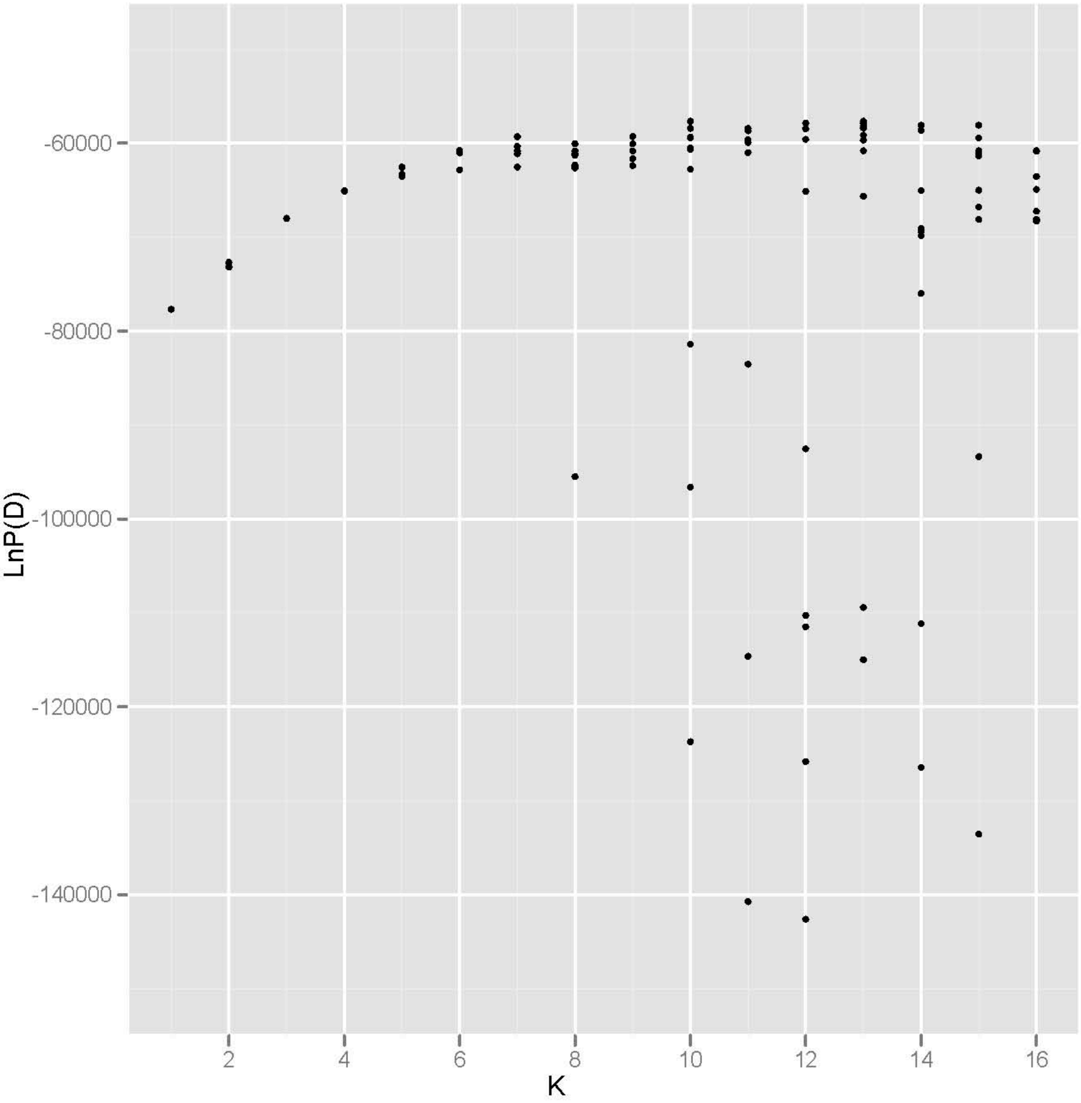

Fig. S3
